# Comprehensive Transcriptomic Analysis Reveals Dysregulated Competing Endogenous RNA Network in Endocrine Resistant Breast Cancer Cells

**DOI:** 10.3389/fonc.2020.600487

**Published:** 2020-11-24

**Authors:** Liang Gao, Kunwei Shen, Ni Yin, Min Jiang

**Affiliations:** ^1^ Institutes of Biology and Medical Sciences, Soochow University, Suzhou, China; ^2^ Comprehensive Breast Health Center, Ruijin Hospital, Shanghai Jiao Tong University School of Medicine, Shanghai, China; ^3^ Department of Oncology, The First Affiliated Hospital of Soochow University, Suzhou, China

**Keywords:** transcriptome profile, endocrine resistance, breast cancer, non-coding RNA, tamoxifen, fulvestrant

## Abstract

**Background:**

Tamoxifen and fulvestrant, both approved for endocrine therapy, have remarkably increased the prognosis of hormone receptor-positive breast cancer patients. However, acquired resistance to endocrine therapy greatly reduces its clinical efficacy. Accumulating evidence suggests a pivotal role of non-coding RNAs (ncRNAs) in breast cancer endocrine resistance, but the specific functions of ncRNAs in tamoxifen and fulvestrant resistance remain largely unknown.

**Methods:**

Microarray analysis was performed for endocrine therapy sensitive (MCF-7), tamoxifen-resistant (LCC2), and dual tamoxifen and fulvestrant-resistant (LCC9) breast cancer cells. Gene ontology and pathway analysis were conducted for functional prediction of the unannotated differentially expressed ncRNAs. Competing endogenous RNA regulatory networks were constructed.

**Results:**

We discovered a total of 3,129 long non-coding RNAs (lncRNAs), 13,556 circular RNAs (circRNAs), 132 microRNAs, and 3358 mRNAs that were significantly differentially expressed. We constructed co-expression networks for lncRNA-mRNA, circRNA-mRNA, and microRNA-mRNA. In addition, we established lncRNA-microRNA-mRNA and circRNA-microRNA-mRNA regulatory networks to depict ncRNA crosstalk and transcriptomic regulation of endocrine resistance.

**Conclusions:**

Our study delineates a comprehensive profiling of ncRNAs in tamoxifen and fulvestrant resistant breast cancer cells, which enriches our understanding of endocrine resistance and sheds new light on identifying novel endocrine resistance biomarkers and potential therapeutic targets to overcome endocrine resistance.

## Introduction

Breast cancer remains one of the leading causes for cancer-related mortality in women, with an incidence rate of 124.7 per 100,000 population and a mortality rate of 20.6 per 100,000 population (1). Around 70% of breast cancers are estrogen receptor (ER) positive, and therefore can be treated with endocrine therapy (2), which exerts anti-tumor activity by depriving cancer cells of growth stimulating hormones. The selective ER modulator tamoxifen and the selective ER degrader fulvestrant are two major treatment options of endocrine therapy. Despite their prominent clinical benefit, the development of drug resistance, in almost all patients with metastases, increases cancer relapse rate and adversely affects the prognosis (2). The mechanisms underlying acquired resistance to tamoxifen and fulvestrant remain poorly understood.

Non-coding RNAs (ncRNAs) are a class of RNAs without protein encoding function but exert essential biological effects at both transcriptional and post-transcriptional levels. The diverse range of ncRNA members includes long ncRNAs (lncRNAs), microRNAs, and circular RNAs (circRNAs). The ncRNAs are extensively transcribed in the human genome, and they exert essential biological functions, though not fully understood (3). To date, accumulating evidence indicates that ncRNAs participate in the regulation of various physiological processes and different pathological conditions, such as inflammation and cancer (4, 5). Moreover, different RNA species, such as lncRNA, circRNA, and mRNA can compete in binding microRNA, thus constituting a competing endogenous RNA (ceRNA) network, in which gene expression is coordinately regulated by different ncRNAs (6).

In recent years, the role of ncRNAs in breast cancer drug resistance has been increasingly illuminated. Both microRNA and lncRNA have been reported to participate in tamoxifen and fulvestrant resistance in breast cancer (7). For example, miR-214 could increase both tamoxifen and fulvestrant sensitivity by targeting UCP2 and inhibiting autophagy in breast cancer cells (8). By contrast, the over-expression of lncRNA H19 augmented autophagy by reducing Beclin1 gene promoter region methylation, which caused tamoxifen resistance in ER-positive breast cancer cells (9). In addition, H19 could prevent ER degradation mediated by fulvestrant, which was related to Notch and c-MET receptor signaling pathway activation (10). Furthermore, the role of circRNA in drug resistance of breast cancer gains increasing attention (11). For example, circ_0025202 has been reported to function as a microRNA sponge for miR-182-5p to regulate FOXO3a activity, thus inhibiting tumor growth and enhancing tamoxifen sensitivity (12). However, evidence of circRNA participation in fulvestrant resistance is still lacking, the long-term use of which is hindered by acquired resistance. Admittedly, previous studies focusing on single ncRNA species sequencing in combination with bioinformatic analysis, or ncRNA interactions such as lncRNA-mRNA and microRNA-mRNA networks, provide information about roles of ncRNAs in endocrine resistance, but these studies are still limiting considering the wide-ranging regulatory crosstalks between different RNA species. Therefore, we aimed to perform a comprehensive transcriptomic analysis of tamoxifen and fulvestrant resistant breast cancer cells, which covers lncRNA, circRNA, microRNA, and mRNA, to help elucidate ncRNA-mediated regulation of endocrine resistance, identify breast cancer endocrine resistance biomarkers, and provide potential targets to overcome drug resistance.

In this study, we examined differentially expressed (DE) ncRNAs in breast cancer cells with acquired resistance to tamoxifen and fulvestrant using microarray analysis. Gene ontology (GO) analysis revealed biological processes, cellular components, and molecular functions that were involved in endocrine resistance. Pathway analysis showed enriched pathways associated with tamoxifen and fulvestrant resistance. According to the ceRNA hypothesis, we established lncRNA-microRNA-mRNA and circRNA-microRNA-mRNA regulatory networks to help elucidate the transcriptomic regulation of endocrine resistance. This is the first study to analyze breast cancer endocrine resistance using comprehensive transcriptomic profiling involving lncRNA, circRNA, microRNA, and mRNA. This study deepens our understanding of tamoxifen and fulvestrant resistance, provides potential drug resistance biomarkers and uncovers possible therapeutic targets for combating endocrine resistance.

## Materials and Methods

### Cell Lines and Reagents

MCF-7 is a human breast cancer cell line. LCC2 and LCC9 are two derivatives of MCF-7 cell line, which are tamoxifen resistant and dual tamoxifen and fulvestrant resistant, respectively. MCF-7, LCC2, and LCC9 were kindly provided by Robert Clarke (Georgetown University Medical Center, Washington, DC, USA). MEM, phenol red free IMEM and FBS were purchased from Gibco. Charcoal-stripped FBS was purchased from Biological Industries. 4-Hydroxytamoxifen and fulvestrant were purchased from Sigma-Aldrich. Methylthiazolyldiphenyl-tetrazolium bromide (MTT) was purchased from Beyotime. PCR primers were synthesized by Sangon Biotech.

### MTT Assay

MCF-7, LCC2, and LCC9 cells were maintained in MEM supplemented with 5% FBS and 1% penicillin/streptomycin in a cell culture incubator at 37°C and 5% CO_2_. Prior to treatment, maintaining medium was changed to phenol red free IMEM supplemented with 5% charcoal-stripped FBS and 1% penicillin/streptomycin (stripped medium). Two days later, cells were digested and resuspended in stripped medium. Then, cells were seeded into 96-well plate at a concentration of 2,500 cells per well (100 µl). One day after seeding, tamoxifen and fulvestrant were diluted with phenol red free IMEM and added to corresponding sample wells. The final concentration gradient for tamoxifen was as the following: 10, 8, 7, 6, 5, 4, 3, 2, 1, and 0 µM. The final concentration gradient for fulvestrant was as the following: 10,000, 1,000, 100, 10, 1, 0.1, 0.01, 0.001, 0.0001, and 0 nM. On day 4, culture medium was aspirated and cells were supplemented with the same fresh medium containing tamoxifen or fulvestrant. After 7 days, cell viability was measured using MTT assay. Briefly, 0.5% MTT solution diluted with fresh culture media was added to each well and cells were incubated at 37°C for another 4 h. Then, supernatant was aspirated and 150 µl DMSO was added. After a 10-min incubation at room temperature with gentle shaking, optical absorbance was read at 490 nm.

### Microarray: Agilent Human lncRNA + mRNA Array V4.0 + CapitalBiotech Human circRNA Array V2.0

The Agilent human lncRNA + mRNA Array V4.0 was used for the profiling of lncRNA and mRNA in MCF-7, LCC2, and LCC9 cell lines. Each array contains probes that could interrogate around 41,000 lncRNAs and 34,000 mRNAs in human genome. The target sequences for these lncRNA and mRNA were derived from a wide range of databases: 23,898 from GENCODE/ENSEMBL, 21,488 from LNCipedia, 14,353 from Human LincRNA Catalog (13), 13,701 from NRED (ncRNA Expression Database), 7,760 from RefSeq, 5,627 from USCS, 3,019 from LncRNAs-a (Enhancer-like), 1,053 from Antisense ncRNA pipeline, 1,038 from H-InvDB, 962 UCRs, 848 from Chen Ruisheng lab (Institute of Biophysics, Chinese Academy of Science), and 407 Hox ncRNAs.

The CapitalBiotech human circRNA Array V2.0 was used for the profiling of circRNA in MCF-7, LCC2, and LCC9 cell lines. Each array contains probes capable of interrogating approximately 170,340 human circRNAs, the target sequences of which are from Circbase, Deepbase and publication of Rybak-Wolf (14). Each circRNA was simultaneously detected by a long probe and a short probe.

### Cell Culture and RNA Extraction

MCF-7, LCC2, and LCC9 cells that were maintained in MEM supplemented with 5% FBS and 1% penicillin/streptomycin were digested, resuspended in stripped medium and seeded in T75 flasks (0.8 million cells/T75 flask). Two days after seeding, the culture medium was aspirated and cells were supplemented with fresh stripped medium. At day 4, culture medium was aspirated and cells were washed with normal saline twice. Then, cells were harvested by incubating with 2 ml Trizol (Invitrogen) for 5 min, with pipetting for cell detachment. Three biological replicates for each cell line were used for the following microarray profiling. Total RNA was extracted from MCF-7, LCC2, and LCC9 breast cancer cells using Trizol (Invitrogen). Extracted RNA was then purified using mirVana microRNA Isolation Kit (Ambion). RNA purity and concentration were determined using spectrophotometer (OD260/280) (NanoDrop ND-1000). RNA integrity was determined using Agilent RNA 6000 Nano kit on 2100 Bioanalyzer instrument (Agilent Technologies). Only RNA with RNA integrity number higher than 6 was used for analysis.

### RNA Amplification, Labeling, and Hybridization

cDNA labeled with fluorescent dye (Cy5 and Cy3-dCTP) was produced using CapitalBio cRNA Amplification and Labeling Kit (CapitalBio). Extracted total RNA was used to generate double-stranded (ds) cDNA using the CbcScript reverse transcriptase with cDNA synthesis system (Capitalbio). dsDNA synthesis was completed with DNA polymerase and RNase H, and then derived dsDNA was purified using NucleoSpin Extract II Kit (Macherey-Nagel) and used as templates for *in vitro* transcription reactions at 37°C for 14 h using T7 Enzyme Mix. The derived RNA was purified using the RNA Clean-up Kit (Macherey-Nagel).

The RNA was then labelled using Klenow enzyme labeling strategy. In brief, amplified RNA (2 μg) was mixed with random nanomer (4 μg), followed by denaturation at 65°C for 5min, and cooling on ice. Afterward, CbcScript II reverse transcriptase (1.5 μl), 4×first-strand buffer (5 μl) and 0.1M DTT (2 μl) were added. The mixture was incubated for 10 min at 25°C, followed by 90 min at 37°C. The cDNA was purified using NucleoSpin Extract II Kit (Macherey-Nagel). The vacuum evaporated cDNA (14 μl) was mixed with random nanomer (4 μg) and heated for 3 min at 95°C, followed by snap cooling on ice for 5 min. Afterward, 5 μl Klenow buffer, Cy5-dCTP or Cy3-dCTP (GE Healthcare, final concentration: 40 μM), and dNTPs (final concentrations: 240 μM dATP, 240 μM dGTP, 240 μM dTTP, 120 μM dCTP) were added. After adding 1.2 μl Klenow enzyme (Takara), the reaction was started and continued for 90 min at 37°C. Labeled cDNA products were purified using PCR NucleoSpin Extract II Kit (Macherey-Nagel). Labeled controls and test samples were dissolved in hybridization solution (80 μl) containing 25% formamide, 0.2% SDS, 5×Denhardt’s solution and 3×SSC. After denaturation at 95°C for 3 min, cDNA was loaded onto a microarray for overnight hybridization in an Agilent Hybridization Oven at 20 rpm and 42°C. The products were washed with two consecutive solutions (0.2% SDS, 2× SSC for 5 min at 42°C, and 0.2× SSC for 5 min at room temperature).

### Microarray Imaging and Data Analysis

The summarization, normalization, and quality control of the microarray data were performed using GeneSpring software V13.0 (Agilent). The threshold for differential expression was a fold change ≥ 2 or ≤−2, in combination with a Benjamini-Hochberg corrected p value of 0.05 for lncRNA and mRNA, or a t-test p value of 0.05 for circRNA. Due to the smaller number of DE microRNAs, a fold change ≥1.5 or ≤−1.5 was used as the threshold (q < 0.05). The data was log_2_ transformed and then median centered by genes with the help of the Adjust Data function in CLUSTER 3.0. After that, the data was analyzed for hierarchical clustering with average linkage (15). Tree visualization was completed using Java Treeview (Stanford University School of Medicine, Stanford, CA, USA).

### Construction of Coding-Noncoding Gene Co-Expression Network and ceRNA Regulatory Network

The coding-noncoding gene co-expression network was established according to the correlation analysis of DE lncRNAs/circRNAs/microRNAs and mRNAs based on their expression levels. The Pearson correlation was calculated for each gene pair. Only pairs with Pearson correlation coefficient ≥ 0.99 and p value < 0.05 were selected to construct the network using Cytoscape. The degree of one gene means the number of links that one gene has to other genes, which reflects gene centrality and relative importance (16).

By using miRanda software to find shared microRNAs between lncRNAs and mRNAs, we established the lncRNA-microRNA-mRNA network on the basis of lncRNA-mRNA correlation network (17). For the circRNA-microRNA-mRNA network, we combined circRNA-mRNA and circRNA-microRNA networks. The circRNAs and mRNAs were positively correlated while both of them were negatively correlated with shared microRNAs.

### Cis- and Trans-Acting lncRNA Prediction

The cis-acting lncRNAs were searched within 10 kb of a protein-coding gene. For target gene prediction by correlation, the cis-acting lncRNA prediction was based on lncRNA correlation to co-expressed protein-coding genes [Pearson’s correlation coefficient ≥ 0.99, adjusted p value < 0.5 (Holm method)]. The trans-acting lncRNA prediction was conducted using BLAT tools (Standalone BLAT) by comparing the lncRNA full sequence with the 3’ UTR of its co-expressed mRNAs. For target gene prediction by correlation, only lncRNA-mRNA pairs with Pearson’s correlation coefficient ≥ 0.99 and adjusted p value < 0.5 (Holm method) were included. The transcription factor prediction was carried out using the sequence spanning from 2000 bp upstream to 500 bp downstream of the transcription start site of lncRNA.

### Gene Ontology Annotations and Pathway Analysis

GO analysis revealed potential functions of DE ncRNAs in three hierarchical aspects: biological processes (BP), cellular components (CC), and molecular function (MF) (18–20). Pathway analysis showed enriched pathways of the DE genes using a combination of databases including KEGG (21–23), PID (24), BioCarta (25), Reactome (26), BioCyc (27, 28), and PANTHER (29, 30).

### Statistical Analysis

Analysis of MTT assay results was performed using Graphpad Prism 8.0. Survival analysis was performed using the TANRIC (The Atlas of non-coding RNA in Cancer) tool (31), and a p value less than 0.05 was regarded as statistically significant.

## Results

### Characterization of Breast Cancer Cell Lines With Acquired Endocrine Resistance

**Figure 1:**
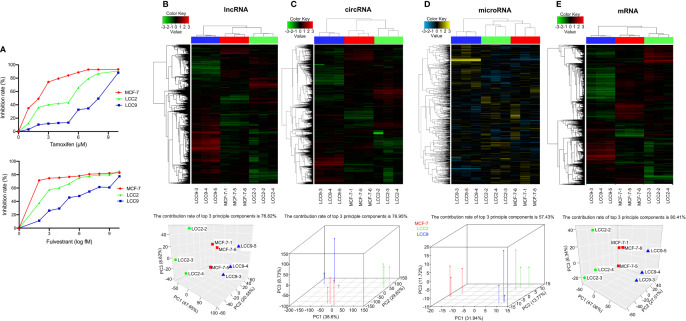
Identification of differentially expressed (DE) ncRNAs in endocrine sensitive and resistant breast cancer cells. **(A)** The sensitivity of MCF-7, LCC2, and LCC9 cells to tamoxifen and fulvestrant. For tamoxifen sensitivity, cells were treated with tamoxifen at the following concentrations: 10, 8, 7, 6, 5, 4, 3, 2, 1, and 0 µM. For fulvestrant sensitivity, cells were treated with fulvestrant at the following concentrations: 10,000, 1,000, 100, 10, 1, 0.1, 0.01, 0.001, 0.0001, and 0 nM. Clustered heatmaps show the profiling for DE lncRNAs **(B)**, circRNAs **(C)**, microRNAs **(D)**, and mRNAs **(E)** (n = 3). Principal component analysis using DE lncRNAs **(B)**, circRNAs **(C)**, microRNAs **(D)**, and mRNAs **(E)** shows optimal separation of the three cell lines. Red, MCF-7; Green, LCC2; Blue, LCC9.

MCF-7 is a human breast cancer cell line that is sensitive to tamoxifen and fulvestrant. LCC2 is a tamoxifen resistant derivative of MCF-7, whereas another derivative, LCC9, is resistant to both tamoxifen and fulvestrant. The three cell lines (MCF-7, LCC2, and LCC9) were treated with serial concentrations of tamoxifen and fulvestrant for 1 week. Then, cell viability was determined using MTT assay (**Figure 1A**). We validated that MCF-7 was sensitive to both tamoxifen and fulvestrant; LCC2 was only sensitive to fulvestrant; LCC9 was resistant to both drugs.

### Identification of Differentially Expressed lncRNAs, circRNAs, microRNAs, and mRNAs

Total RNA was extracted from MCF-7, LCC2, and LCC9 cells for transcriptome analysis (three biological replicates per cell line). Analysis of microarray data revealed numerous DE lncRNAs, circRNAs and mRNAs (fold change ≥2 or ≤−2, q < 0.05). Due to the smaller number of DE microRNAs, a fold change ≥1.5 or ≤−1.5 was used as the threshold (q < 0.05). The profiling for DE lncRNAs, circRNAs, microRNAs and mRNAs were illustrated using clustered heatmaps in **Figures 1B–E**. Principal component analysis plots were placed next to heatmaps, which showed optimal separation of the three cell lines used in the experiment (**Figures 1B–E**).

To explore the role of ncRNAs in endocrine resistance, we set three comparison groups: LCC2vsMCF-7 (tamoxifen resistance), LCC9vsMCF-7 (tamoxifen and fulvestrant resistance), and LCC2mergeLCC9 (merged ncRNAs between LCC2vsMCF-7 and LCC9vsMCF-7). The merged ncRNAs are more likely to contribute to tamoxifen resistance, whereas the remaining unmerged ncRNAs in the LCC9vsMCF-7 group may be involved in acquired fulvestrant resistance. Volcano plots showed the pattern of up-regulated and down-regulated lncRNAs, circRNAs, microRNAs and mRNAs (**Supplementary Figure 1**).

When comparing LCC2 to MCF-7 cells, there were 921 DE lncRNAs (459 up-regulated and 462 down-regulated), 6,860 DE circRNAs (2,222 up-regulated and 4,638 down-regulated), 25 DE microRNAs (19 up-regulated and 6 down-regulated), and 1,213 DE mRNAs (530 up-regulated and 683 down-regulated). When comparing LCC9 to MCF-7 cells, there were 2,498 DE lncRNAs (1,895 up-regulated and 603 down-regulated), 8,279 DE circRNAs (5,431 up-regulated and 2,848 down-regulated), 119 DE microRNAs (99 up-regulated and 20 down-regulated), and 2,603 DE mRNAs (1,181 up-regulated and 1,422 down-regulated). When DE ncRNAs were merged between LCC2vsMCF-7 and LCC9vsMCF-7, we obtained a group of DE ncRNAs that were more likely to confer tamoxifen resistance (LCC2mergeLCC9). In this group, there were 290 DE lncRNAs (156 up-regulated and 134 down-regulated), 1,583 DE circRNAs (740 up-regulated and 843 down-regulated), 12 DE microRNAs (9 up-regulated and 3 down-regulated), and 458 DE mRNAs (185 up-regulated and 273 down-regulated). The top 10 up-regulated and 10 down-regulated lncRNAs, circRNAs, microRNAs and mRNAs for the three comparison groups were listed in **Supplementary Table 1**. To validate our microarray results, we performed quantitative real-time PCR to analyze the expression levels of selected ncRNAs. The quantitative real-time PCR results were consistent with our microarray findings (data not shown).

**Figure 2:**
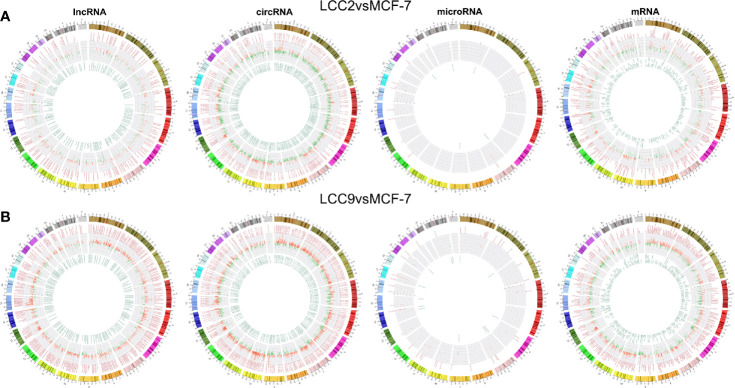
Chromosomal distribution of differentially expressed ncRNAs in LCC2vsMCF-7 **(A)** and LCC9vsMCF-7 **(B)**. The outer circle represents differentially colored chromosomes. The up-regulated and down-regulated ncRNAs are shown in red and green in middle and inner circles, respectively. Each line represents one RNA and the height indicates its degree of differential expression.

Chromosomal distribution of DE LncRNAs was shown in **Figures 2A–B**. Subgroup analysis (**Supplementary Figure 2**) indicated that the percentage for intergenic, antisense, divergent, intronic and sense DE lncRNAs in group LCC2vsMCF-7 were 49.2%, 27.3%, 5.1%, 3.5%, and 2.1%, respectively. The percentage for intergenic, antisense, intronic, divergent and sense DE lncRNAs in group LCC9vsMCF-7 were 47%, 27.6%, 5.9%, 3.5%, and 2.7%, respectively. The percentage for intergenic, antisense, intronic, divergent and sense DE lncRNAs in group LCC2mergeLCC9 were 53.1%, 26.9%, 1%, 3.8%, and 1.7%, respectively. Chromosomal distribution of DE circRNAs, microRNAs, and mRNAs were shown in circos plots (**Figures 2A–B**).

### lncRNA-mRNA Co-Expression Network and Functional Prediction

**Figure 3:**
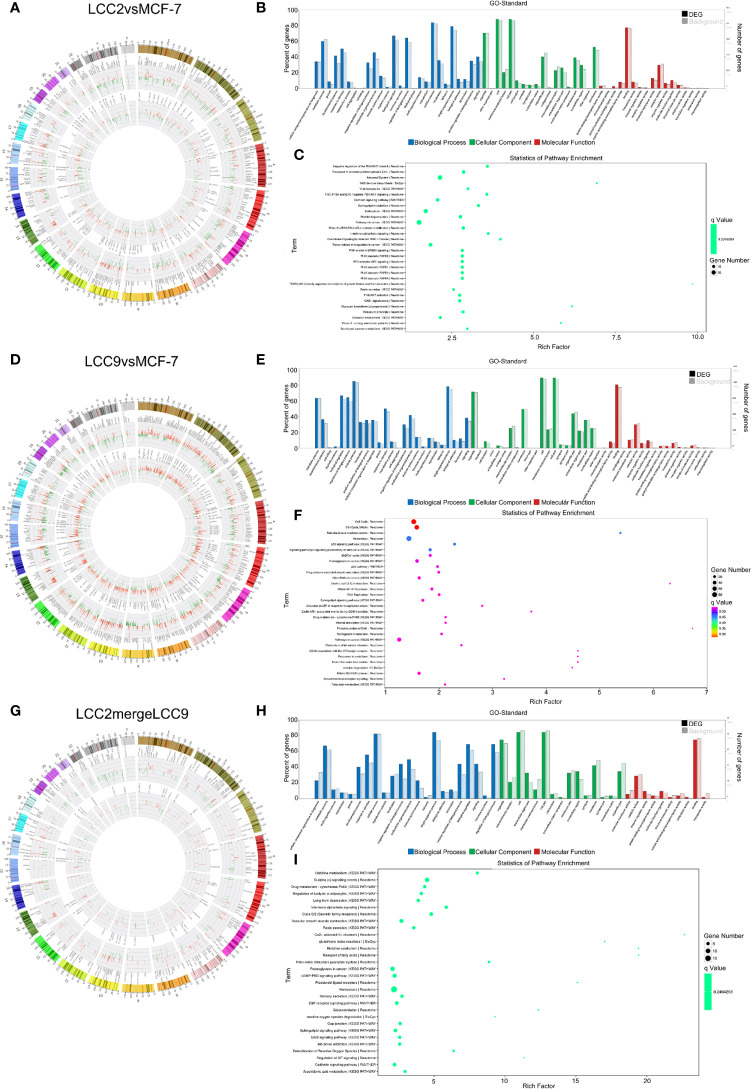
The lncRNA-mRNA co-expression network and functional prediction. Chromosomal distribution of up-regulated (red) and down-regulated (green) lncRNAs (inner circles) and mRNAs (middle circles) for LCC2vsMCF-7 **(A)**, LCC9vsMCF-7 **(D)**, and LCC2mergeLCC9 **(G)** are shown in circos plots. **(B, E, H)** Gene Ontology (GO) analysis of genes encoding differentially expressed (DE) mRNAs in the lncRNA-mRNA co-expression network. Blue, biological processes (BP); green, cellular components (CC); red, molecular functions (MF). Bars with solid color and diagonal stripes indicate the number of genes annotated to different GO terms in the DE gene set and background gene set, respectively. **(C, F, I)** Pathway analysis of genes encoding DE mRNAs in the lncRNA-mRNA co-expression network. The size of the circle denotes the number of genes enriched in the pathway whereas different colors indicate their different q values.

Correlation analysis was performed between lncRNAs and mRNAs. The chromosomal distribution of up-regulated and down-regulated lncRNAs and mRNAs was shown in circos plots (**Figures 3A, D, G**). The lncRNAs with highest degree in the co-expression network were mostly unannotated, such as ENST00000513165.1 and NR_109925.1 in the LCC2vsMCF-7 group, uc.226-, TCONS_00003759, and uc021pna.1 in the LCC9vsMCF-7 group, as well as uc004fgb.3 and ENST00000555023.1 in the LCC2mergeLCC9 group. GO and pathway analysis was performed for genes encoding DE mRNAs in the lncRNA-mRNA network to help reveal lncRNA functions. In GO analysis, genes were classified according to GO terms: BP, MF and CC (**Figures 3B, E, H**). We found some enriched and meaningful GO terms that are worth further study. For example, lipid metabolism, one of the most enriched BP terms in LCC2mergeLCC9, is indicated to be related to endocrine resistance by earlier studies (32). Another example would be the protein serine/threonine kinase inhibitor activity, which is one of the most enriched MF terms in the LCC9vsMCF-7 group. Given the established role of serine/threonine kinases in endocrine resistance (33), it is worthwhile to investigate the altered kinase inhibitor activity in endocrine resistant cells. The top 30 most enriched GO terms were shown in **Supplementary Figure 3**. Pathway analysis was performed using a combination of data sources from KEGG pathway, PID, BioCarta, Reactome, BioCyc, and PANTHER. The top 30 enriched pathways were shown in **Figures 3C, F, I**. The representative enriched pathways for LCC2vsMCF-7 included PI3K/AKT signaling network, cadherin signaling pathway and pathways in cancer. For LCC9vsMCF-7, the significantly enriched pathways of interest included cell cycle, p53 signaling pathway and stem cell pluripotency regulation pathways. Some of these pathways are already known to participate in endocrine resistance. For example, p53 protein accumulation was found to be related to endocrine resistance in breast cancer patients, and it reduced post-relapse survival (34). In LCC2mergeLCC9, the most enriched pathways were histidine metabolism, G alpha (s) signaling events and drug metabolism-cytochrome P450. Of note, cytochrome P450 (CYP) 2D6 could affect tamoxifen metabolism, revealed by genetic polymorphism analysis of 730 breast cancer patients in the PHACS study (35). Therefore, the drug metabolism-cytochrome P450 pathway is of clinical importance and further study may discover ncRNAs associated with tamoxifen metabolism and resistance. **Supplementary Figure 4** displayed the number of genes enriched in second-class KEGG pathways.

Target prediction of lncRNAs by correlation and differential expression was also performed. lncRNAs can regulate neighboring and remote genes in cis- and trans-mechanisms. A cis-acting lncRNA is defined as a lncRNA regulating protein-coding genes within the proximity of its genomic locus (10 kb in our case). Cis-regulation can be categorized into sense, antisense, intergenic, intronic and bidirectional regulations. In contrast, trans-acting lncRNAs can regulate gene expression not in proximity to its site of synthesis. Trans-regulation is complicated and also not well understood, which includes chromatin state and gene expression regulation, nuclear structure and organization modification, as well as interaction with other proteins and RNAs (36). In our case, we performed trans-prediction by comparing the full sequence of the lncRNA with the 3’ UTR of its co-expressed mRNAs using the BLAT tool, which represents one mechanism of trans-regulation: microRNA sequestration. The networks of lncRNA and predicted target genes for three comparison groups were displayed in **Supplementary Figure 5**. GO and pathway analysis was performed for target genes which were predicted based on differential expression. The number of genes enriched in GO terms was shown in **Supplementary Figure 6**. Pathway analysis results were shown in **Supplementary Figure 6**. One of the most enriched GO terms was the positive regulation of toll-like receptor signaling pathway. Previous evidence indicates that Toll-like receptor 4 contributes to the development of paclitaxel resistance in advanced breast cancer cells by increasing the expression of pro-inflammatory cytokines such as IL-6 and IL-8, as well as the anti-apoptotic protein XIAP (37). Whether it also contributes to endocrine resistance is unclear and further study of the relevant terms revealed in our study may identify novel mechanisms.

**Figure 4:**
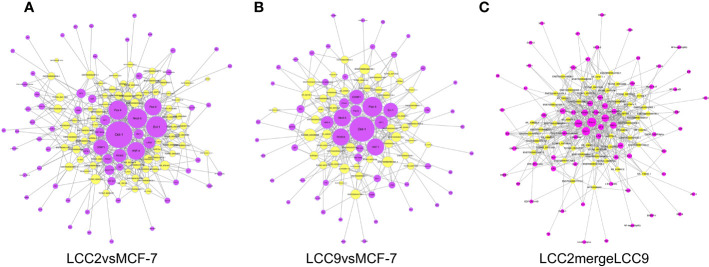
Networks of lncRNAs (yellow) and predicted interacting transcription factors (purple) for LCC2vsMCF-7 **(A)**, LCC9vsMCF-7 **(B)**, and LCC2mergeLCC9 **(C)**.

Of note, lncRNAs could regulate target gene expression by interaction with transcription factors. The transcription factor prediction was carried out using the sequence from 2,000 bp upstream to 500 bp downstream of the transcription start site of lncRNA. We analyzed transcription factors that might be involved in lncRNA mediated regulation of gene expression (**Figure 4**). In the network of lncRNAs and transcription factors, the transcription factors with highest degree in group LCC2vsMCF-7 were Oct-1, Evi-1, Nkx2-5, and Pax-4, all of which were correlated to multiple lncRNAs. Interestingly, overexpression of Evi-1 was observed in basal subtype, ER negative breast cancers and related to poor prognosis (38). In addition, Evi-1 was involved in the proliferation, apoptosis and epithelial-mesenchymal transition (EMT) in breast cancer stem cells (39). Given the tumor promoting role of Evi-1 in the above studies, whether it participates in endocrine resistance is worth further research. For LCC9vsMCF-7, the transcription factors with highest degree were Oct-1, Pax-4, HNF-1, Nkx2-5, FOXD3, and Evi-1. In LCC2mergeLCC9 group, transcription factors with highest degree were Pax-4, Oct-1, COMP1, Nkx2-5, HNF-4, and FOXD3. Of note, FOXD3 has tumor suppressive role in breast cancer. Its down-regulation was associated with lymph node metastases in invasive ductal carcinomas (40) and EMT in breast cancer (41).

### circRNA-mRNA Co-Expression Network and Functional Prediction

**Figure 5:**
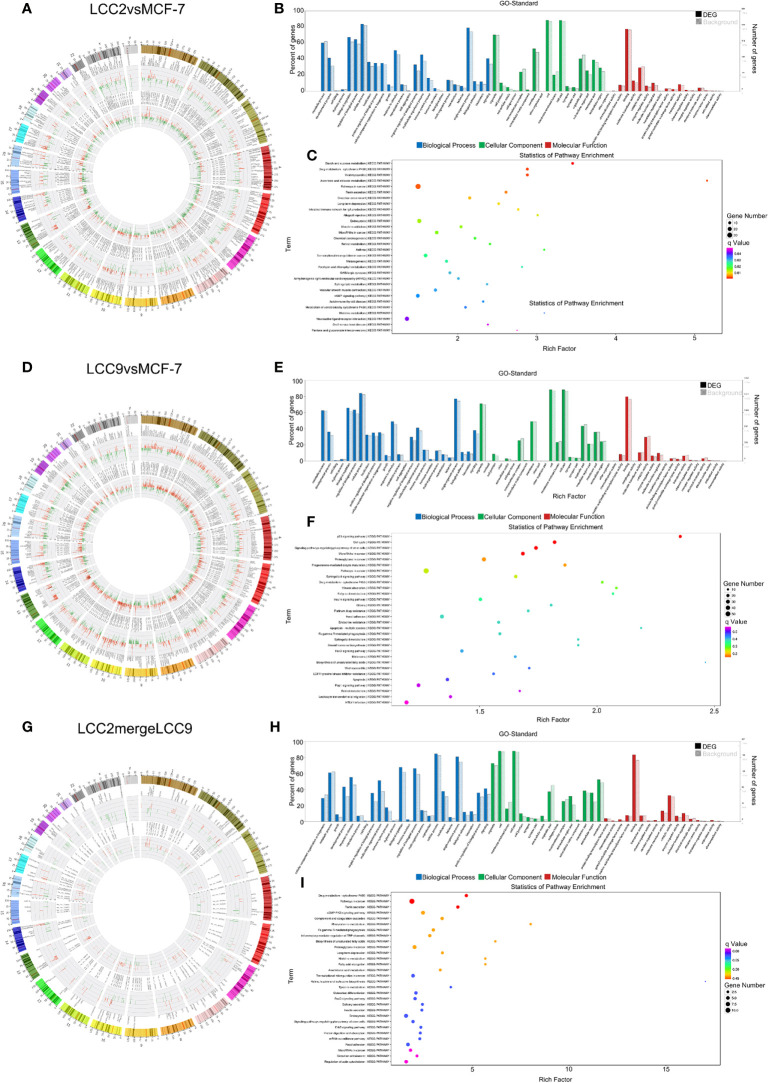
The circRNA-mRNA co-expression network and functional prediction. Chromosomal distribution of up-regulated (red) and down-regulated (green) circRNAs (inner circles) and mRNAs (middle circles) for LCC2vsMCF-7 **(A)**, LCC9vsMCF-7 **(D)**, and LCC2mergeLCC9 **(G)** are shown in circos plots. **(B, E, H)** Gene Ontology (GO) analysis of genes encoding differentially expressed (DE) mRNAs in the circRNA-mRNA co-expression network. Blue, biological processes (BP); green, cellular components (CC); red, molecular functions (MF). Bars with solid color and diagonal stripes indicate the number of genes annotated to different GO terms in the DE gene set and background gene set, respectively. **(C, F, I)** Pathway analysis of genes encoding DE mRNAs in the circRNA-mRNA co-expression network. The size of the circle denotes the number of genes enriched in the pathway whereas different colors indicate their different q values.

The chromosomal distribution of up-regulated and down-regulated circRNAs and mRNAs was shown in circos plots (**Figures 5A, D, G**). circRNA-mRNA correlation network for LCC2mergeLCC9 was shown in **Supplementary Figure 7**. The circRNAs with highest degree in the co-expression network were mostly unannotated, including hsa_circ_0105926, hsa_circ_0040835, and hsa_circ_0077827 in the LCC2vsMCF-7 group, hsa-circRNA2454-19 and hsa-circRNA10089-3 in the LCC9vsMCF-7 group, as well as hsa_circRNA7619-30 and hsa_circ_0114066 in the LCC2mergeLCC9 group. GO and pathway analysis was performed for genes encoding DE mRNAs in circRNA-mRNA networks to help reveal novel circRNA functions (**Figures 5B, C, E, F, H, I**). In the LCC2vsMCF-7 group, the most enriched BP terms were related to development and oxygen-containing compound response; the top 3 enriched MF terms were receptor binding, peptidase regulator activity and peptidase inhibitor activity. In LCC9vsMCF-7, the most enriched BP terms were associated with development, epithelial cell proliferation and cell adhesion, whereas the most enriched MF terms included protein kinase inhibitor activity. Of note, Notch signaling pathway was found to be a highly enriched BP term in LCC2mergeLCC9. In fact, the role of Notch signaling pathway in endocrine resistance has been established before. Tamoxifen or fulvestrant treatment could increase breast cancer stem cell activity *via* Notch signaling pathway, resulting in endocrine resistance. In addition, Notch inhibitors could overcome tamoxifen resistance in ER positive breast cancers (42). Top 30 enriched GO terms for the three comparison groups were listed in **Supplementary Figure 8**. The representative enriched pathways for LCC2vsMCF-7 include drug metabolism - cytochrome P450 and pathways in cancer. For LCC9vsMCF-7, the significantly enriched pathways include p53 signaling pathway, cell cycle, signaling pathways regulating pluripotency of stem cells, microRNAs in cancer and proteoglycans in cancer. Consistent with our data, the role of cancer stem cell in tamoxifen resistance has been reported previously. The expression level of the stem cell marker SOX2 was higher in tamoxifen resistant cell lines and patient tumors after endocrine therapy failure, and SOX2 silencing could restore tamoxifen sensitivity (43). The significantly enriched pathways for LCC2mergeLCC9 include drug metabolism-cytochrome P450, pathways in cancer, and cGMP-PKG signaling pathway. The number of genes enriched in second-class KEGG pathways was shown in **Supplementary Figure 9**.

### microRNA-mRNA Co-Expression Network and Functional Prediction

**Figure 6:**
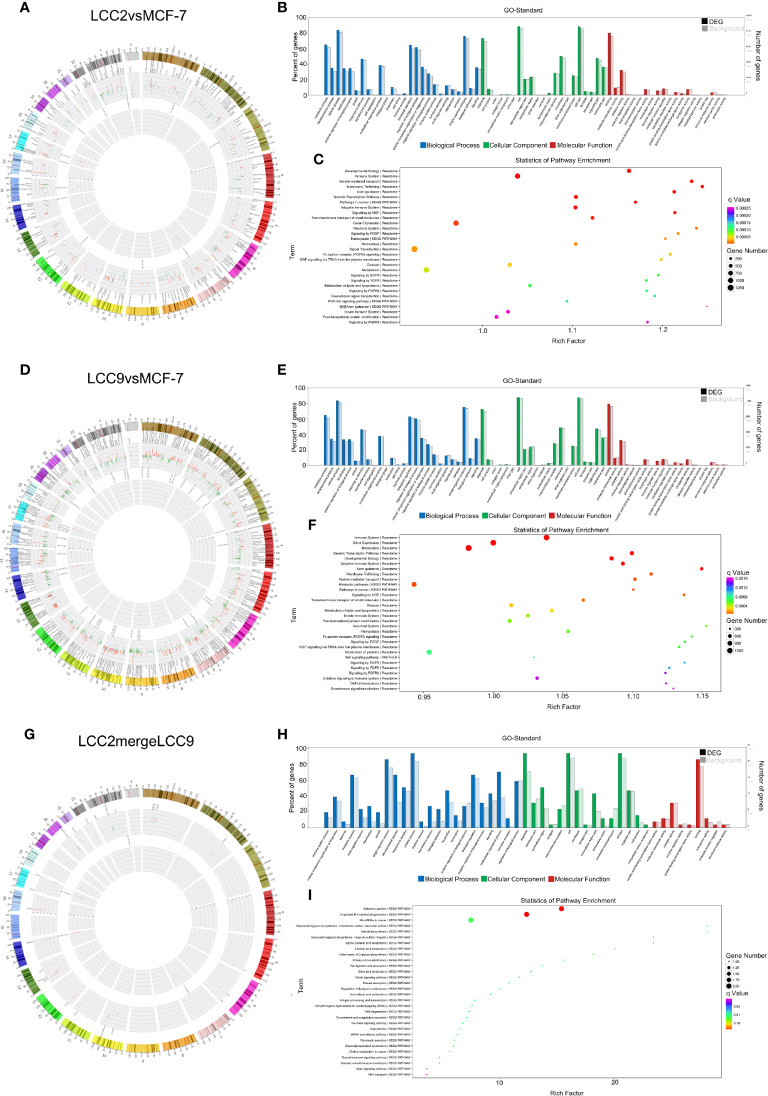
The microRNA-mRNA co-expression network and functional prediction. Chromosomal distribution of up-regulated (red) and down-regulated (green) microRNAs (inner circles) and mRNAs (middle circles) for LCC2vsMCF-7 **(A)**, LCC9vsMCF-7 **(D)**, and LCC2mergeLCC9 **(G)** are shown in circos plots. **(B, E, H)** Gene Ontology (GO) analysis of genes encoding differentially expressed (DE) mRNAs in the microRNA-mRNA co-expression network. Blue, biological processes (BP); green, cellular components (CC); red, molecular functions (MF). Bars with solid color and diagonal stripes indicate the number of genes annotated to different GO terms in the DE gene set and background gene set, respectively. **(C, F, I)** Pathway analysis of genes encoding DE mRNAs in the microRNA-mRNA co-expression network. The size of the circle denotes the number of genes enriched in the pathway whereas different colors indicate their different q values.

Correlation networks were also constructed to show the negative correlation between microRNAs and their target mRNAs (**Supplementary Figure 10**). Chromosomal distribution of up-regulated and down-regulated microRNAs and their target mRNAs was shown in **Figures 6A, D, G**. For LCC2vsMCF-7, the microRNAs with highest degree included the up-regulated hsa-miR-409-3p and hsa-miR-195−5p, as well as the down-regulated hsa-miR-1299 and hsa-miR-205-5p. For LCC9vsMCF-7, a number of microRNAs had a high degree including the up-regulated hsa-miR-485-5p, hsa-miR-495-3p, hsa-miR-370-3p, hsa-miR-125b-5p, hsa-miR-432-5p, hsa-miR-205-5p, and hsa-miR-10a-5p, as well as the down-regulated hsa-miR-27b-3p and hsa-miR-23b-3p. In the LCC2mergeLCC9 group, the microRNAs in the correlation network were all up-regulated, including hsa-miR-105-5p, hsa-miR-10a-5p, hsa-miR-767-5p, hsa-miR-409-3p, hsa-miR-489-3p, and hsa-miR-134-5p. Among these DE microRNAs, miR-134-5p, hsa-miR-125b-5p, and hsa-miR-10a-5p were significantly associated with short relapse-free time in hormone receptor positive breast cancer, which supports the role of these microRNAs in cancer recurrence and tamoxifen resistance (44).

In order to determine functions of DE microRNAs, we carried out GO and KEGG pathway analysis of the genes targeted by DE microRNAs. Candidate genes were classified using the GO terms (**Figures 6B, E, H**), with top 30 enriched terms shown in **Supplementary Figure 11**, including cell communication regulation and signaling regulation. **Figures 6C, F, I** showed pathway analysis results of these DE genes. Among them, immune system, membrane trafficking and adherens junction were representative enriched pathways that may be involved in endocrine resistance. The number of genes enriched in second-class KEGG pathways was shown in **Supplementary Figure 12**.

### Construction of Competing Endogenous RNA Regulatory Network

Competing endogenous RNAs (ceRNA) are a class of RNAs with microRNA recognition elements (MREs) that could compete for binding microRNAs (6). MREs function as a type of RNA language for different RNA species, such as mRNA, lncRNA and circRNA, to communicate with each other for the regulation of gene expression. Regulatory ceRNA network analysis was performed to assess the functions and underlying molecular mechanisms of ncRNAs in acquired resistance to tamoxifen and fulvestrant in breast cancer.

#### lncRNA-microRNA-mRNA Regulatory Network

**Figure 7:**
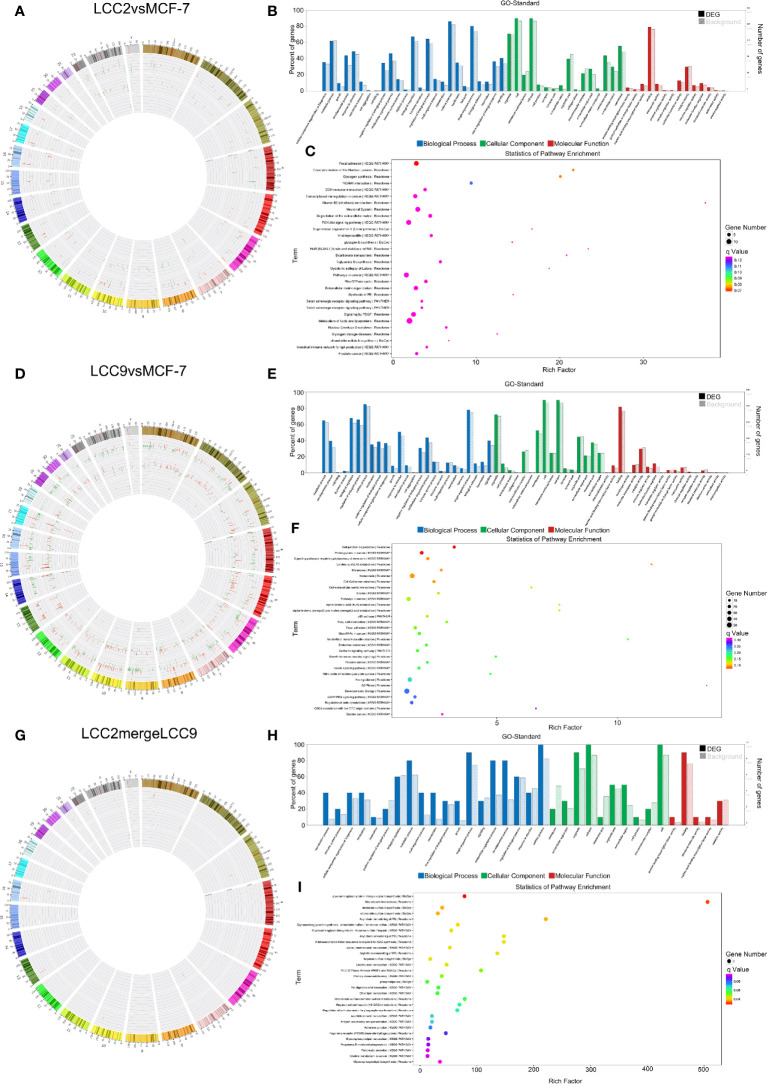
The lncRNA-microRNA-mRNA regulatory network. Chromosomal distribution of up-regulated (red) and down-regulated (green) mRNAs, lncRNAs and microRNAs (outer to inner) for LCC2vsMCF-7 **(A)**, LCC9vsMCF-7 **(D)**, and LCC2mergeLCC9 **(G)** are shown in circos plots. **(B, E, H)** Gene Ontology (GO) analysis of genes encoding differentially expressed (DE) mRNAs in the lncRNA-microRNA-mRNA network. Blue, biological processes (BP); green, cellular components (CC); red, molecular functions (MF). Bars with solid color and diagonal stripes indicate the number of genes annotated to different GO terms in the DE gene set and background gene set, respectively. **(C, F, I)** Pathway analysis of genes encoding DE mRNAs in the lncRNA-microRNA-mRNA network. The size of the circle denotes the number of genes enriched in the pathway whereas different colors indicate their different q values.

**Figure 8:**
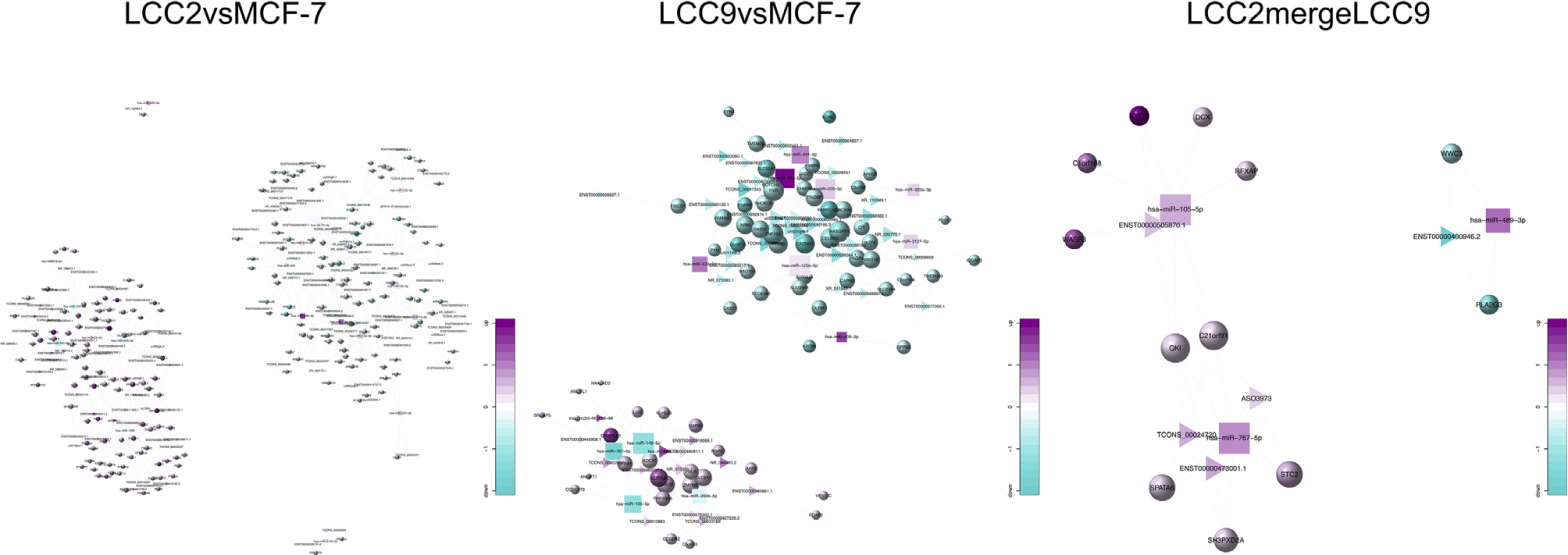
The lncRNA-microRNA-mRNA networks for the three comparison groups. Circles, triangles and squares represent mRNAs, lncRNAs and microRNAs, respectively. The node size indicates node degree whereas node color denotes the degree of differential expression.

The lncRNA-microRNA-mRNA network was constructed on the basis of lncRNA-mRNA correlation network by using miRanda software to find shared microRNAs between lncRNAs and mRNAs (17). The chromosomal distribution of up-regulated and down-regulated lncRNA, microRNA and mRNA was shown in the circos plots for all three comparison groups (**Figures 7A, D, G** **)**. The lncRNA-microRNA-mRNA networks were shown in **Figure 8**. A microRNA could be shared by multiple lncRNAs and mRNAs. In the LCC2vsMCF-7 group, such microRNAs with high degree include the up-regulated hsa-let-7b-5p, hsa-miR-134-5p, hsa-miR-10a-5p, and hsa-miR-489-3p, as well as the down-regulated hsa-miR-1299, hsa-miR-205-5p, and hsa-miR-767-5p. In the LCC9vsMCF-7 group, shared microRNAs with high degree include the up-regulated hsa-miR-10a-5p, hsa-miR-125b-5p, hsa-miR-205-5p, and hsa-miR-433-3p, as well as the down-regulated hsa-miR-149-5p, hsa-miR-767-5p, hsa-miR-105-5p, and hsa-miR-200b-5p. In the merged group, such microRNAs include the up-regulated hsa-miR-105-5p, hsa-miR-767-5p, and hsa-miR-489-3p. Interestingly, some of these microRNAs overlapped with those found in the microRNA-mRNA correlation network, such as miR-134-5p, hsa-miR-125b-5p, and hsa-miR-10a-5p, which were related to relapse in tamoxifen-treated hormone receptor positive breast cancer (44).

To help elucidate the biological role of ncRNA interactions within the ceRNA network, the genes encoding DE mRNAs in the lncRNA-microRNA-mRNA network were subjected to GO and pathway analysis (**Figures 7B, C, E, F, H, I**). Interestingly, development and morphogenesis were highly enriched BP terms in both LCC2vsMCF-7 and LCC9vsMCF-7. Some other GO terms also attracted our attention, such as hippo signaling regulation, which is one of the most enriched BP terms in LCC2mergeLCC9. Earlier study indicates that the hippo signaling pathway could modulate ERα activity (45), and further investigations are needed to fully understand the role of hippo signaling pathway in endocrine resistance. Top 30 enriched GO terms for the three comparison groups were listed in **Supplementary Figure 13**. The representative significantly enriched pathways for LCC2vsMCF-7 were related to focal adhesion, ECM-receptor interaction and transcriptional mis-regulation in cancer. The significantly enriched pathways for LCC9vsMCF-7 included cell junction organization, proteoglycans in cancer, and signaling pathways regulating pluripotency of stem cells. The noteworthy enriched pathways for LCC2mergeLCC9 were about biosynthesis of glycoaminoglycan-protein linkage region and glycosaminoglycan. The role of glycosaminoglycan in breast cancer is noteworthy, which has started to attract oncologists’ attention. For example, heparan sulfate is one type of glycosaminoglycan. Heparan sulfate degradation was increased in the presence of overexpressed heparanase induced by tamoxifen treatment in ER positive breast cancer, which may be able to promote tumor invasion and therefore confer tamoxifen resistance (46). In addition, the expression of syndecan-1, which has heparan sulfate chains and is able to promote cancer invasiveness, could be induced by ER suppression using selective ER down-regulators in breast cancer cells (47). It is possible that syndcan-1, with its glycosaminoglycan chains, accounts for endocrine resistance in breast cancer. This demonstrates the value of further investigation into our transcriptomic profiling study. **Figures 7C, F, I** displayed pathway analysis of these DE genes. Enriched pathways included focal adhesion, cell junction organization, proteoglycans in cancer, and stem cell pluripotency regulation. The number of genes enriched in second-class KEGG pathways was shown in **Supplementary Figure 14**.

#### circRNA-microRNA-mRNA Regulatory Network

**Figure 9:**
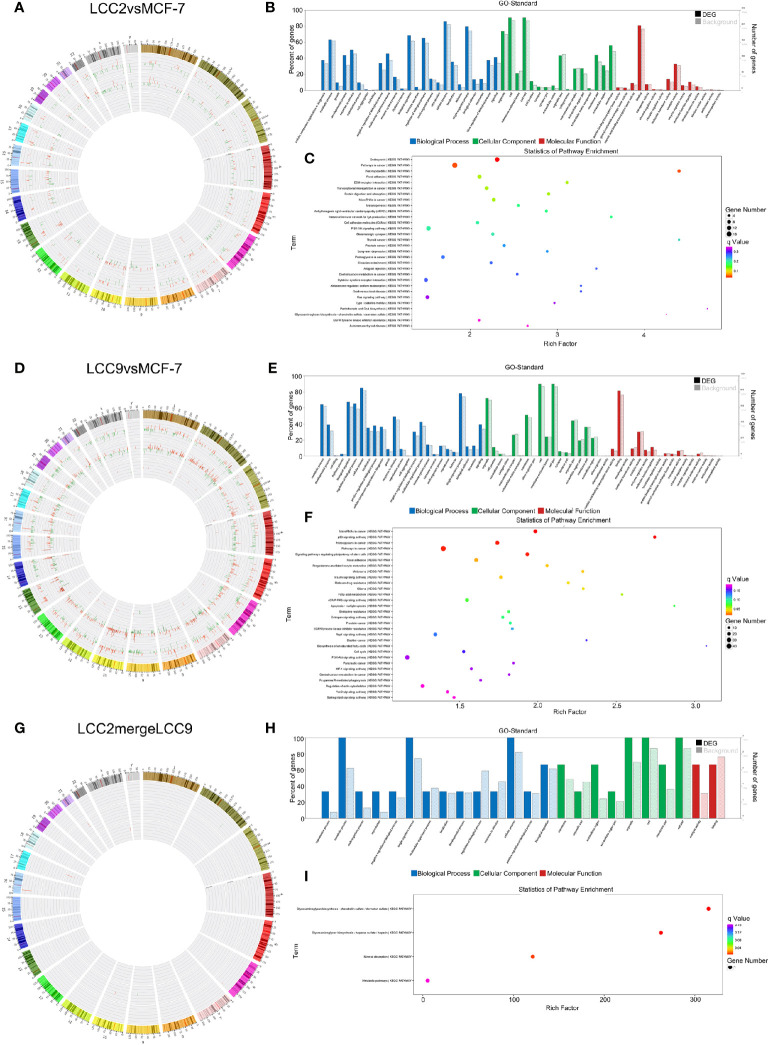
The circRNA-microRNA-mRNA regulatory network. Chromosomal distribution of up-regulated (red) and down-regulated (green) mRNAs, circRNAs and microRNAs (outer to inner) for LCC2vsMCF-7 **(A)**, LCC9vsMCF-7 **(D)**, and LCC2mergeLCC9 **(G)** are shown in circos plots. **(B, E, H)** Gene Ontology (GO) analysis of genes encoding differentially expressed (DE) mRNAs in the circRNA-microRNA-mRNA network. Blue, biological processes (BP); green, cellular components (CC); red, molecular functions (MF). Bars with solid color and diagonal stripes indicate the number of genes annotated to different GO terms in the DE gene set and background gene set, respectively. **(C, F, I)** Pathway analysis of genes encoding DE mRNAs in the circRNA-microRNA-mRNA network. The size of the circle denotes the number of genes enriched in the pathway whereas different colors indicate their different q values.

The circRNA-microRNA-mRNA network was constructed on the basis of circRNA-mRNA and circRNA-microRNA networks. The chromosomal distribution of up-regulated and down-regulated circRNA, microRNA and mRNA was shown in the circos plots (**Figures 9A, D, G**). The microRNAs shared by a number of circRNAs and mRNAs in LCC2vsMCF-7 group included the up-regulated hsa-miR-134-5p, hsa-let-7b-5p, hsa-miR-409-3p, and hsa-miR-10a-5p, as well as the down-regulated hsa-miR-1299, hsa-miR-205-5p, and hsa-miR-767-5p. Previously, it is found that miR-409-3p has tumor suppressive role by binding to 3’ UTR of Akt1 and mediating its down-regulation (48). Given the role of its target gene Akt1 in tamoxifen resistance (49) as well as our ceRNA network analysis results, it is highly possible that miR-409-3p participates in tamoxifen resistance. The shared microRNAs in the LCC9vsMCF-7 group included the up-regulated hsa-miR-370-3p, hsa-miR-432-5p, hsa-miR-485-5p, and hsa-miR-1301-3p, as well as the down-regulated hsa-miR-149-5p, hsa-miR-27b-3p, and hsa-miR-767-5p. Consistent with our results, hsa-miR-27b-3p was reduced in breast cancer tissues from tamoxifen resistant patients when compared with untreated patients, and increasing hsa-miR-27b-3p level could restore tamoxifen sensitivity both *in vitro* and *in vivo* (50). In LCC2mergeLCC9 group, such microRNAs included hsa-miR-767-5p, hsa-miR-489-3p and hsa-miR-10a-5p. Interestingly, hsa-miR-134-5p and hsa-miR-10a-5p were two microRNAs identified in both microRNA-mRNA correlation network and lncRNA-microRNA-mRNA network. In combination with previous study indicating their role in tamoxifen resistance of hormone receptor positive breast cancer (44), it is worthwhile to study the circRNAs associated with these two microRNAs that were identified in this ceRNA network.

To help elucidate functions of unannotated circRNAs and associated RNA interactions in endocrine resistance, the genes encoding DE mRNAs in the circRNA-microRNA-mRNA network were subjected to GO and pathway analysis (**Figures 9B, C, E, F, H, I**). In LCC2vsMCF-7, the most enriched BP terms were related to development and response to oxygen-containing compound; the most enriched MF terms were about tensile strength, transcription factor activity and receptor binding. In LCC9vsMCF-7, the most enriched BP terms were related to development and cell proliferation; the most enriched MF terms were associated with enzyme binding, molecular function regulator and kinase binding; the most enriched CC term was cell junction. In LCC2mergeLCC9, the most enriched BP terms were related to mRNA and RNA destabilization and glycosaminoglycan biosynthesis; the most enriched MF terms were about ferric-chelate reductase activity and xylosyltransferase activity. Top 30 enriched GO terms for the three comparison groups were listed in **Supplementary Figure 15**. The noteworthy enriched pathways for LCC2vsMCF-7 included pathways in cancer, focal adhesion, ECM-receptor interaction and transcriptional mis-regulation in cancer. For LCC9vsMCF-7, the enriched pathways included microRNAs in cancer, p53 signaling pathway and proteoglycans in cancer. The significantly enriched pathways for LCC2mergeLCC9 were related to glycosaminoglycan biosynthesis and mineral absorption. The role of glycoproteins in endocrine resistance is being widely studied. For example, the transmembrane glycoprotein CD44 was up-regulated in tamoxifen-resistant breast cancer cells and enhanced their sensitivity to ErbB ligands and hyaluronan, thus promoting an adverse phenotype (51). The number of genes enriched in second-class KEGG pathways was shown in **Supplementary Figure 16**.

**Figure 10:**
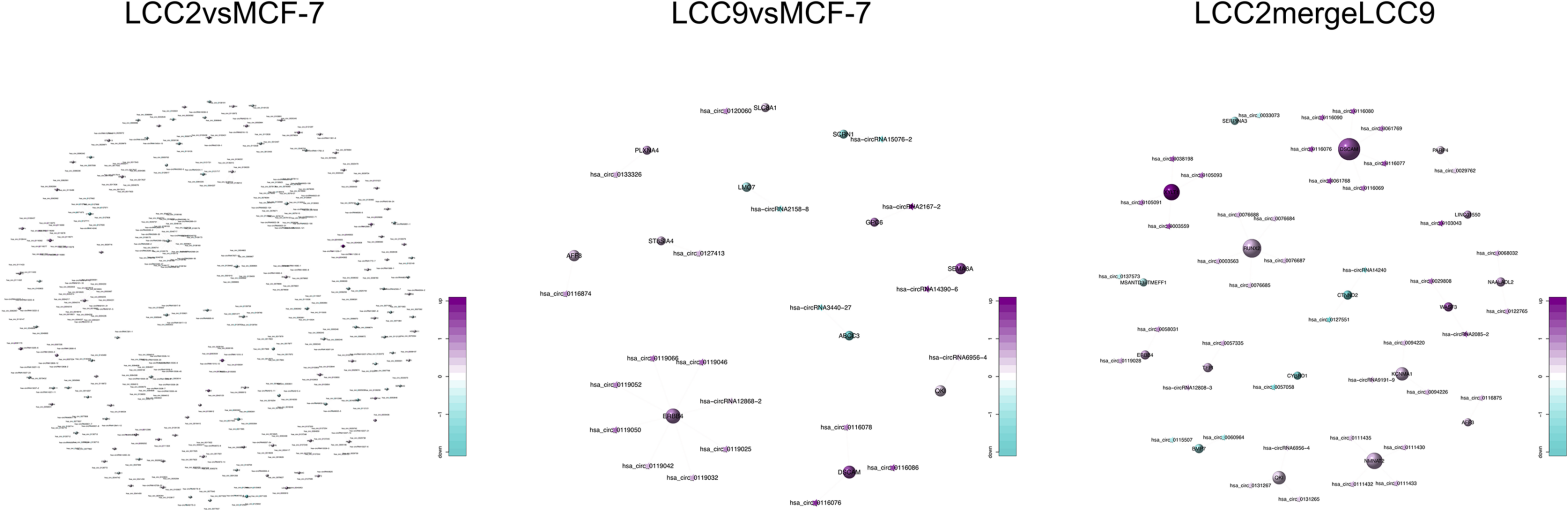
The circRNA-mRNA cis regulation networks for the three comparison groups. Circles and stars represent mRNAs and circRNAs, respectively. The node size indicates node degree whereas node color denotes the degree of differential expression.

Using the genomic location information of circRNAs and mRNAs, we built a circRNA-mRNA cis regulation network (**Figure 10**). GO and KEGG analysis was also performed for genes encoding these cis-regulated mRNAs (**Supplementary Figure 17**). In the LCC2vsMCF-7 group, representative enriched GO terms included enzyme linked receptor protein signaling pathway, transmembrane receptor protein tyrosine kinase signaling pathway and positive regulation of phosphatidylinositol 3-kinase activity. Enriched pathways included central carbon metabolism and microRNAs in cancer. For the LCC9vsMCF-7 group, the most enriched GO terms were related to development and cell junction. Enriched pathways were about fatty acid biosynthesis and metabolism, microRNAs and central carbon metabolism in cancer. In the LCC2mergeLCC9 group, the most enriched GO terms were related to morphogenesis and development. The most noteworthy enriched pathways were related to glycosaminoglycan biosynthesis.

## Discussion

Tamoxifen and fulvestrant are two clinically effective endocrine therapy agents in the treatment of hormone receptor positive breast cancer. However, their clinical benefits are greatly hindered by the development of endocrine resistance. The mechanisms of endocrine resistance are multifaceted and not well understood, among which the role of ncRNAs is gaining increasing attention. In this study, we provide comprehensive profiling of the transcriptome involving lncRNA, circRNA, microRNA, and mRNA in endocrine resistant breast cancer cells. Among our findings, some are consistent with previous discovered molecular targets or pathways in endocrine resistance, while a larger proportion of ncRNAs are unannotated and need further investigation. Our study sheds new light on the understanding of acquired resistance to tamoxifen and fulvestrant, and aids in identifying biomarkers for drug resistance, potential therapeutic targets and prognostic indicators of breast cancer.

Our results were consistent with previous datasets about transcriptomic analysis of endocrine-resistance breast cancers. For example, some pathways are found to be activated in LCC2 cells both by previous dataset (52) (data accessible at NCBI GEO database, accession GSE118774) and our microarray analysis, which include cell cycle, PI3K signaling, p53 signaling, and adherens junction. We also found increased expression of SOX2 in tamoxifen-resistant cells, and this is consistent with another dataset GSE55343, which is focused on mRNA and small RNA (data accessible at NCBI GEO database, accession GSE55343). The up-regulation of the transcription factor SOX2 was validated in tamoxifen resistant cell lines and endocrine therapy ineffective patient tumors (43). SOX2 silencing by siRNA, or SOX2 down-regulation by increasing its transcription repressor MSX2 using a neddylation inhibitor pevonedistat, could restore tamoxifen sensitivity (43, 53). In addition to the above tamoxifen-related studies, we also found molecular targets and pathways in fulvestrant resistance that were consistent with previous discoveries. Notch signaling pathway is one of the most enriched altered pathways we identified in our study. Previously, it’s reported that tumor-bearing mice treated with fulvestrant exhibited enriched self-renewing CD133^hi^ cancer stem cells, in which Notch3 and mRNA transcripts of Notch signaling pathway were elevated. Notch3 reduction by stable short hairpin RNA restored their fulvestrant sensitivity (54). Furthermore, increase of Notch1 activity marker was associated with worse clinical outcome in tamoxifen treated breast cancer patients, according to a meta-analysis of 458 women patients (55). Therefore, further study of ncRNAs associated with the Notch pathway may enrich our understanding of endocrine resistance and identify novel molecular targets for Notch pathway inhibition and endocrine sensitivity enhancement. The consistency with the previous studies indicates the effectiveness of our study. However, the majority of ncRNAs identified are functionally unannotated, which are worth further research.

**Figure 11:**
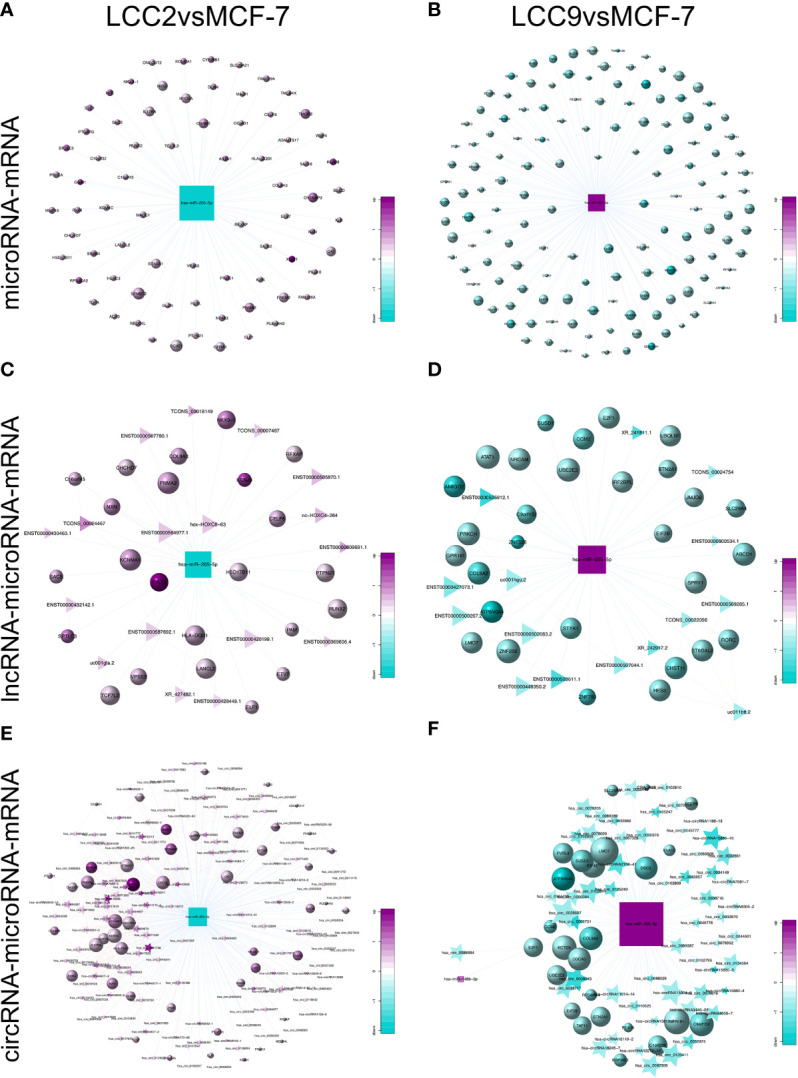
Expression pattern of miR-205-5p and its role in competing endogenous RNA network. The subnetworks of the down-regulated miR-205-5p in LCC2vsMCF-7 include microRNA-mRNA **(A)**, lncRNA-microRNA-mRNA **(C)**, and circRNA-microRNA-mRNA **(E)** networks. The subnetworks of the up-regulated miR-205-5p in LCC9vsMCF-7 include microRNA-mRNA **(B)**, lncRNA-microRNA-mRNA **(D)**, and circRNA-microRNA-mRNA **(F)** networks. Circles, triangles, stars and squares represent mRNAs, lncRNAs, circRNAs, and microRNAs, respectively. The node size indicates node degree whereas node color denotes the degree of differential expression.

To help reveal functional roles of unannotated ncRNAs, we constructed dysregulated ceRNA networks in tamoxifen and fulvestrant resistant breast cancer cells. In a ceRNA network, one microRNA is shared by multiple lncRNAs, circRNAs, and mRNAs as MRE is present in different RNA species for them to crosstalk with each other. Some novel interactions were discovered in our networks. The tumor suppressor miR-205-5p, which was down-regulated in breast cancers, could inhibit breast cancer cell growth and invasion (56). miR-205-5p participated in cell proliferation and EMT by interaction with various targeted genes such as ErbB3, VEGF-A, and ZEB1 (56, 57). On the other hand, there are studies indicating that up-regulation of miR-205-5p in breast cancer cells suppressed the expression of ErbB2 and induced resistance to targeted therapy (58). Moreover, up-regulated miR-205-5p was found to be involved in EMT of breast cancer cells, and miR-205-5p silencing reduced tumor growth and metastasis in mice (59). Such kind of controversy was also seen in our study. We found that the expression of miR-205-5p was reduced when comparing tamoxifen resistant LCC2 cells to parental MCF-7 cells (**Figure 11A**), whereas the level of miR-205-5p increased in tamoxifen and fulvestrant cross-resistant LCC9 cells (**Figure 11B**). Such kind of pattern was also seen when establishing the ceRNA network (**Figures 11C–F**). We could find overlapping genes targeted by miR-205-5p in the microRNA-mRNA, lncRNA-microRNA-mRNA, and circRNA-microRNA-mRNA networks for LCC2vsMCF-7, such as VPS53, NKX3-1, and HLA-DQB1. This also happened in LCC9vsMCF-7, but there were rarely overlapping genes between the two comparison groups. The role of miR-205-5p in endocrine resistance remains elusive. Moreover, given its different expression pattern in tamoxifen and dual resistant cells, its role in tamoxifen and fulvestrant resistance may be different.

**Figure 12:**
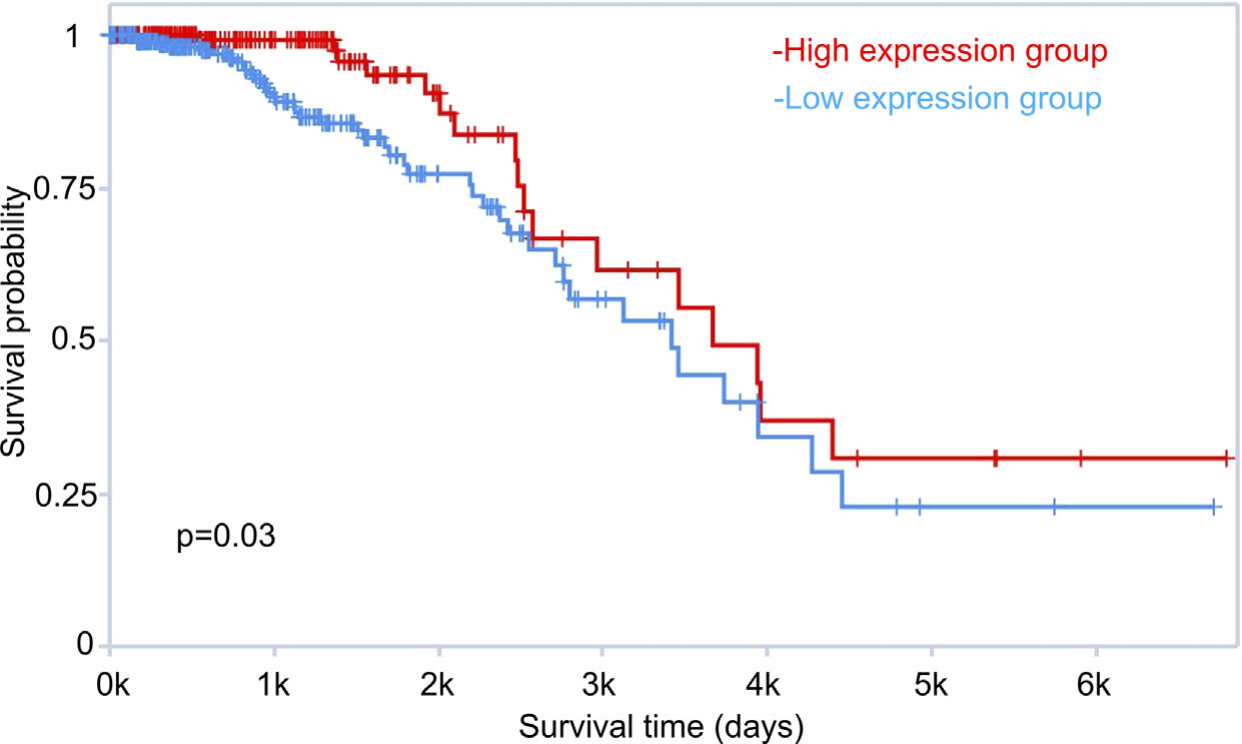
Role of LINC00221 in breast cancer patient survival. Survival analysis of estrogen receptor positive breast cancer patients with high and low LINC00221 expression was performed using TANRIC (The Atlas of non-coding RNA in Cancer, http://bioinformatics.mdanderson.org/main/TANRIC:Overview).

Our comprehensive transcriptomic profiling of tamoxifen and fulvestrant resistant breast cancer cells also revealed potential prognostic markers for ER positive breast cancer patients. We performed survival analysis using the TANRIC database (31). For example, LINC00221 was one of the most down-regulated lncRNAs in the LCC2vsMCF7 group (**Supplementary Table 1**). TANRIC analysis indicated that ER positive breast cancer patients with higher expression of LINC00221 have a higher survival probability (p = 0.03) (**Figure 12**). So far, there is no research about the role of LINC00221 in breast cancer. Therefore, further research is warranted to explore the function of LINC00221 and other ncRNAs revealed by our study that are associated with patient outcome.

## Conclusion

This is the first comprehensive analysis of ceRNA network comprising lncRNA, circRNA, microRNA, and mRNA in tamoxifen and fulvestrant resistant breast cancer cells. The ceRNA networks shed new light on identifying novel biomarkers of endocrine resistance and potential therapeutic targets for overcoming endocrine resistance in breast cancer.

## Data Availability Statement

The datasets presented in this study can be found in online repositories. The names of the repository/repositories and accession number(s) can be found below: GEO GSE159981.

## Author Contributions

MJ and NY designed the study. LG and MJ performed the experiments, analyzed the data, and wrote the manuscript. MJ and NY revised the manuscript. MJ performed the experiments during the revision stage. KS contributed to data interpretation and revised the manuscript during the revision stage. All authors contributed to the article and approved the submitted version.

## Funding

This work was supported by: National Natural Science Foundation of China (81402176, 81402093, 81472296), Natural Science Foundation of Jiangsu Province, China (BK20140288), Postgraduate Research & Practice Innovation Program of Jiangsu Province (KYCX17_2036), Science Technology Project of Suzhou Xiang cheng District (XJ201456, XJ201532), Livelihood Science and Technology of Soochow (SYS201752, SS2018062), and Industry-university-research cooperation, prospective joint research project of Jiangsu Province (BY2015039-01). The funding bodies didn’t influence study design, data collection, analysis, interpretation, and manuscript writing.

## Conflict of Interest

The authors declare that the research was conducted in the absence of any commercial or financial relationships that could be construed as a potential conflict of interest.

## Abbreviations

ER, estrogen receptor; ncRNAs, non-coding RNAs; lncRNA, long non-coding RNA circRNA, circular RNA; ceRNA, competing endogenous RNA; DE, differentially expressed; GO, Gene Ontology; EMT, epithelial-mesenchymal transition; MRE, microRNA recognition element.
