# Supplementary material for: Comprehensive Transcriptomic Analysis Reveals Dysregulated Competing Endogenous RNA Network in Endocrine Resistant Breast Cancer Cells
Source: Front Oncol. 2020 Nov 24;10:600487. doi: 10.3389/fonc.2020.600487 (PMC7723334; doi:10.3389/fonc.2020.600487)
Supplement: Supplementary file 1 [file DataSheet_1.docx]

**Supplementary Figure 1.** Volcano plots showing differentially expressed RNAs when comparing LCC2 (A) and LCC9 (B) cells to parental MCF-7 cells. Red and green dots represent up-regulated and down-regulated RNAs, respectively.

**Supplementary Figure 2.** Subgroup analysis of differentially expressed lncRNAs. The percentages of intergenic, antisense, divergent, intronic and sense differentially expressed lncRNAs in LCC2vsMCF-7 (A), LCC9vsMCF-7 (B) and LCC2mergeLCC9 (C) are shown.

**Supplementary Figure 3.** List of the top 30 most enriched Gene Ontology terms for the genes encoding differentially expressed mRNAs in the lncRNA-mRNA co-expression network. Blue: biological processes (BP); yellow: cellular components (CC); red: molecular functions (MF).

**Supplementary Figure 4.** List of the number of genes enriched in second-class KEGG pathways for the genes encoding differentially expressed mRNAs in the lncRNA-mRNA co-expression network.

**Supplementary Figure 5.** Networks of lncRNAs and their target genes predicted by correlation (A, B, C) and differential expression (D, E, F) for the three comparison groups. Circles and triangles represent genes and lncRNAs, respectively. The node size indicates node degree whereas node color denotes the degree of differential expression.

**Supplementary Figure 6.** Gene Ontology (GO) and pathway analysis of target genes of lncRNAs predicted by differential expression in the three comparison groups. Blue: biological processes (BP); green: cellular components (CC); red: molecular functions (MF). Bars with solid color and diagonal stripes indicate the number of genes annotated to different GO terms in the DE gene set and background gene set, respectively. The size of the circle denotes the number of genes enriched in the pathway whereas different colors indicate their different q values.

**Supplementary Figure 7.** The circRNA-mRNA co-expression network for the LCC2mergeLCC9 group. Circles and stars represent mRNAs and circRNAs, respectively. The node size indicates node degree whereas node color denotes the degree of differential expression.

**Supplementary Figure 8.** List of the top 30 most enriched Gene Ontology terms for the genes encoding differentially expressed mRNAs in the circRNA-mRNA co-expression network. Blue: biological processes (BP); yellow: cellular components (CC); red: molecular functions (MF).

**Supplementary Figure 9.** List of the number of genes enriched in second-class KEGG pathways for the genes encoding differentially expressed mRNAs in the circRNA-mRNA co-expression network.

**Supplementary Figure 10.** Co-expression networks showing the negative correlation between microRNAs and mRNAs for LCC2vsMCF-7 and LCC2mergeLCC9. The network for LCC9vsMCF-7 is not shown due to the huge number of pairs. Circles and squares represent mRNAs and microRNAs, respectively. The node size indicates node degree whereas node color denotes the degree of differential expression.

**Supplementary Figure 11.** List of the top 30 most enriched Gene Ontology terms for the genes encoding differentially expressed mRNAs in the microRNA-mRNA co-expression network. Blue: biological processes (BP); yellow: cellular components (CC); red: molecular functions (MF).

**Supplementary Figure 12.** List of the number of genes enriched in second-class KEGG pathways for the genes encoding differentially expressed mRNAs in the microRNA-mRNA co-expression network.

**Supplementary Figure 13.** List of the top 30 most enriched Gene Ontology terms for the genes encoding differentially expressed mRNAs in the lncRNA-microRNA-mRNA network. Blue: biological processes (BP); yellow: cellular components (CC); red: molecular functions (MF).

**Supplementary Figure 14.** List of the number of genes enriched in second-class KEGG pathways for the genes encoding differentially expressed mRNAs in the lncRNA-microRNA-mRNA network.

**Supplementary Figure 15.** List of the top 30 most enriched Gene Ontology terms for the genes encoding differentially expressed mRNAs in the circRNA-microRNA-mRNA network. Blue: biological processes (BP); yellow: cellular components (CC); red: molecular functions (MF).

**Supplementary Figure 16.** List of the number of genes enriched in second-class KEGG pathways for the genes encoding differentially expressed mRNAs in the circRNA-microRNA-mRNA network.

**Supplementary Figure 17.** Gene Ontology (GO) and KEGG pathway analysis for the genes encoding differentially expressed mRNAs in the circRNA-mRNA cis regulation networks of three comparison groups. Blue: biological processes (BP); green: cellular components (CC); red: molecular functions (MF). Bars with solid color and diagonal stripes indicate number of genes annotated to different GO terms in the DE gene set and background gene set, respectively. The size of the circle denotes number of genes enriched in the pathway whereas different colors indicate their different q values.

**Supplementary Table 1.** List of the top 10 most up-regulated and 10 most down-regulated lncRNAs, circRNAs, microRNAs and mRNAs for the three comparison groups.
