# Supplementary figures and images for: Comprehensive Transcriptomic Analysis Reveals Dysregulated Competing Endogenous RNA Network in Endocrine Resistant Breast Cancer Cells
Source: Front Oncol. 2020 Nov 24;10:600487. doi: 10.3389/fonc.2020.600487 (PMC7723334; doi:10.3389/fonc.2020.600487)

**A****LCC2vsMCF-7****lncRNA****circRNA****microRNA****mRNA**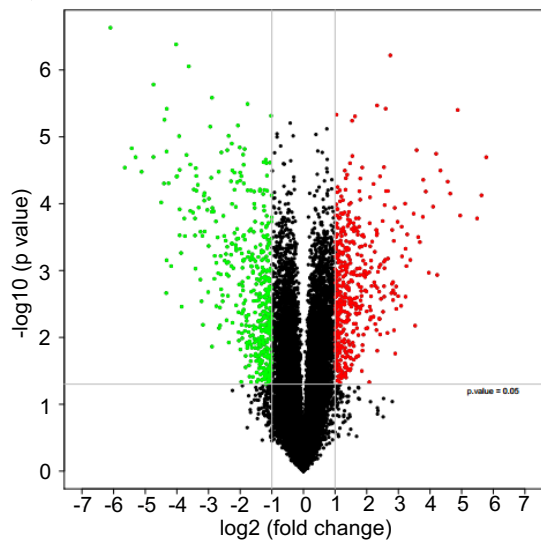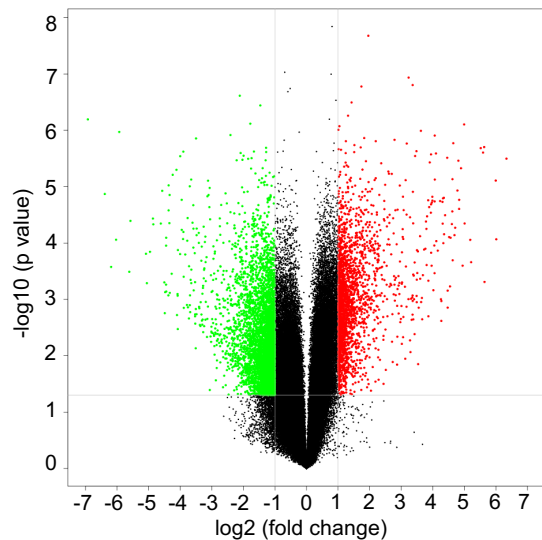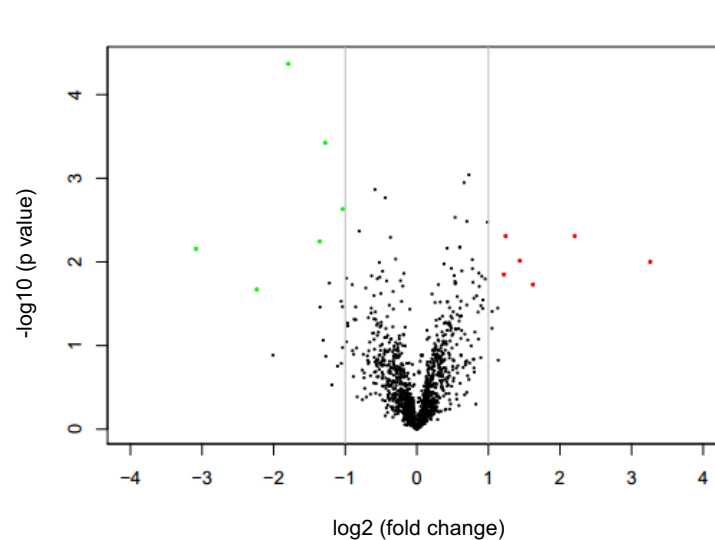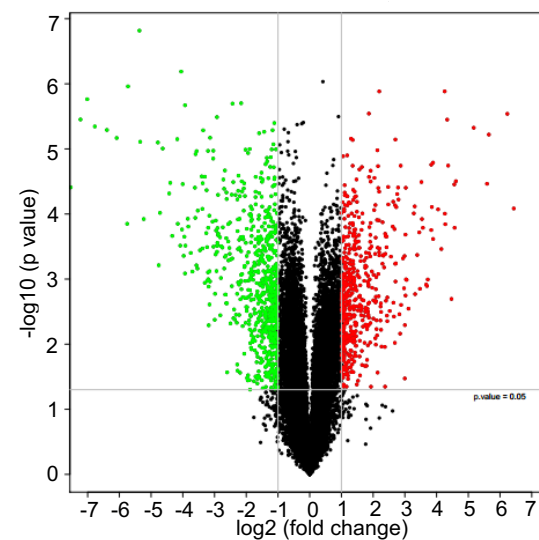**B****LCC9vsMCF-7****Volcano Plot**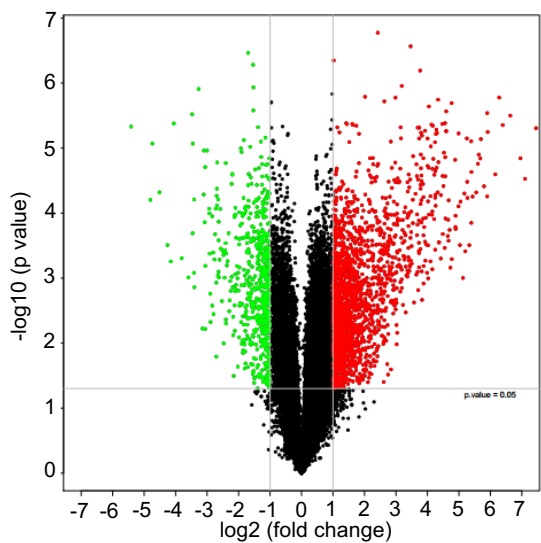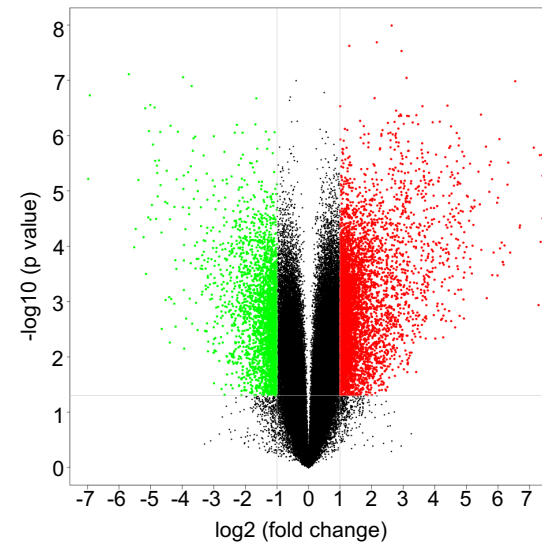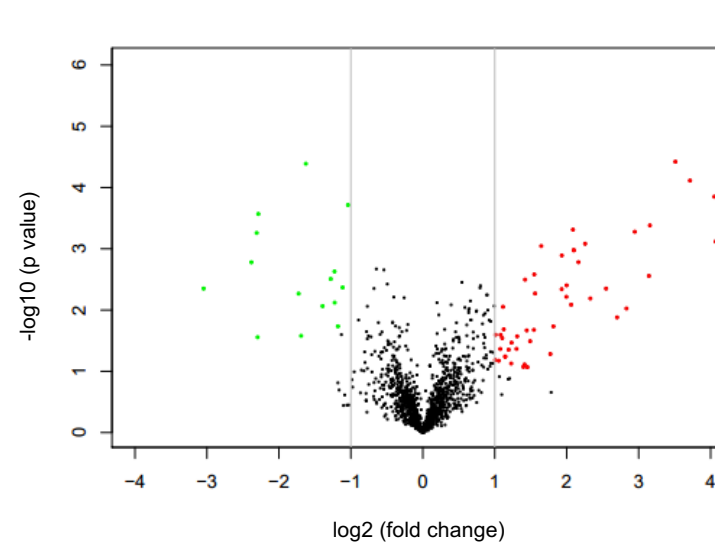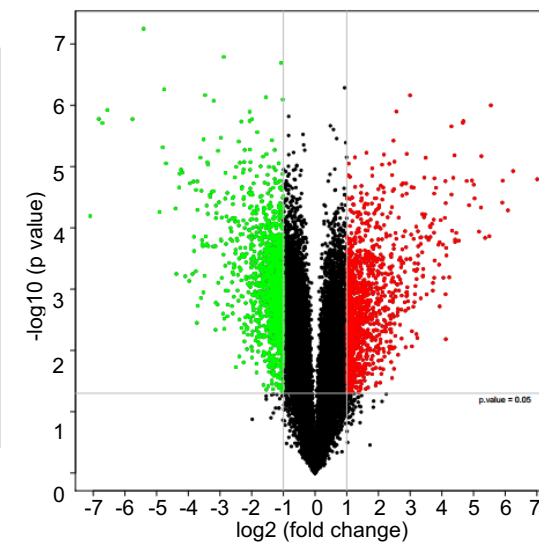

Supplement: Supplementary file 2 [file DataSheet_2.zip › Supplementary Figures_1/Supplementary Figure 1.PDF]

LCC2vsMCF-7

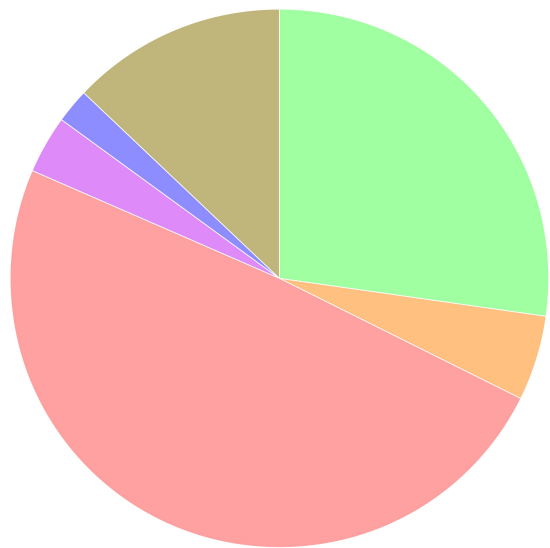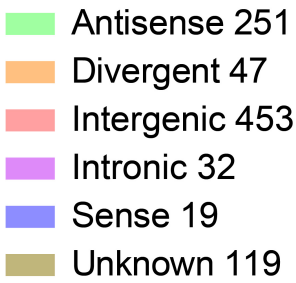

Total=921

LCC9vsMCF-7

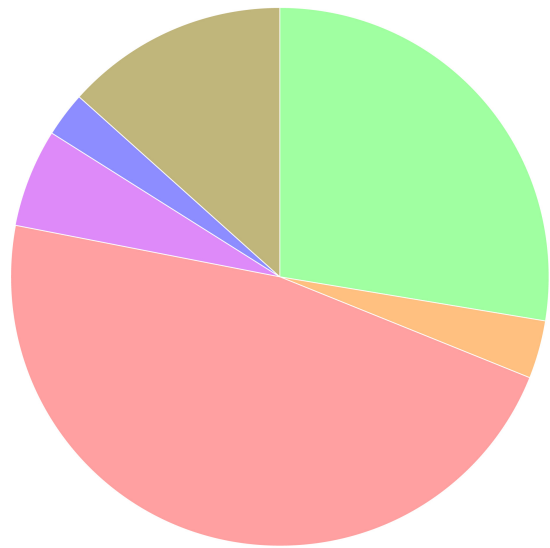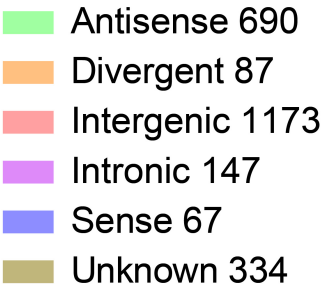

Total=2498

LCC2mergeLCC9

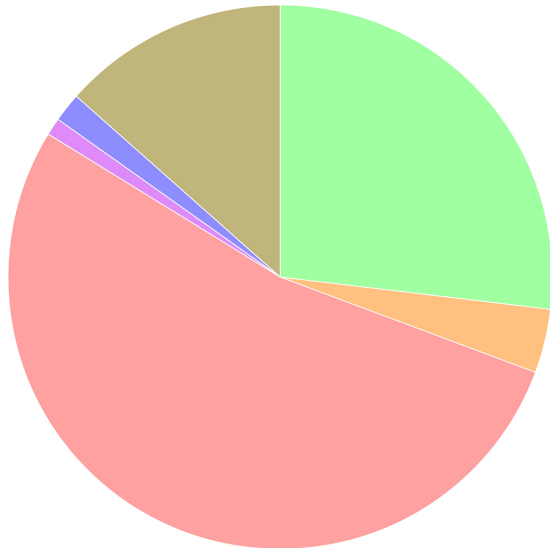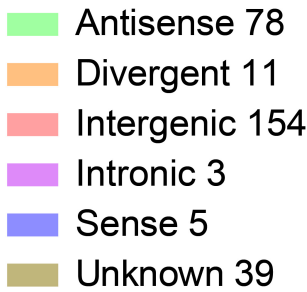

Total=290

Supplement: Supplementary file 2 [file DataSheet_2.zip › Supplementary Figures_1/Supplementary Figure 2.PDF]

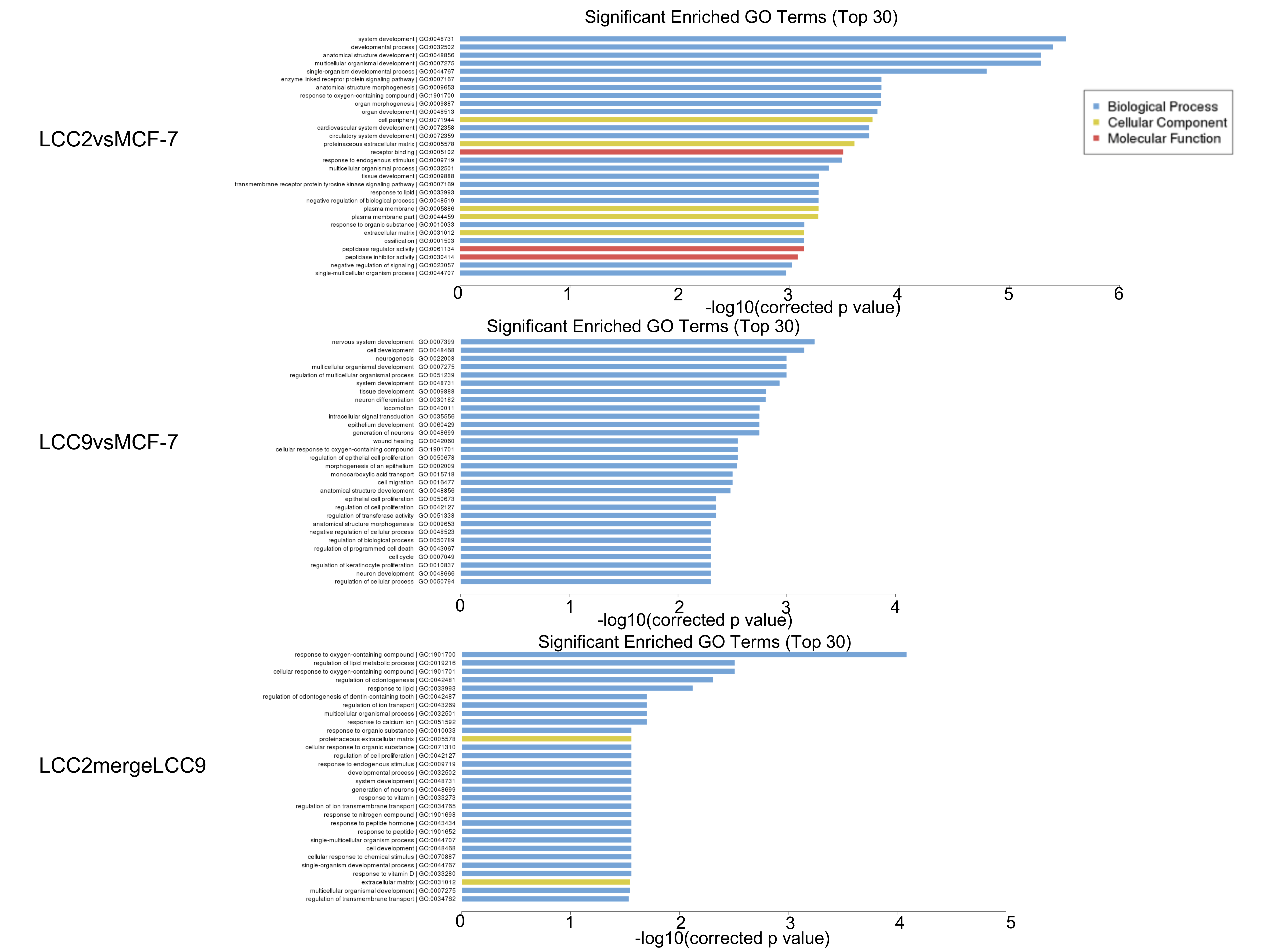

Supplement: Supplementary file 2 [file DataSheet_2.zip › Supplementary Figures_1/Supplementary Figure 3.JPEG]

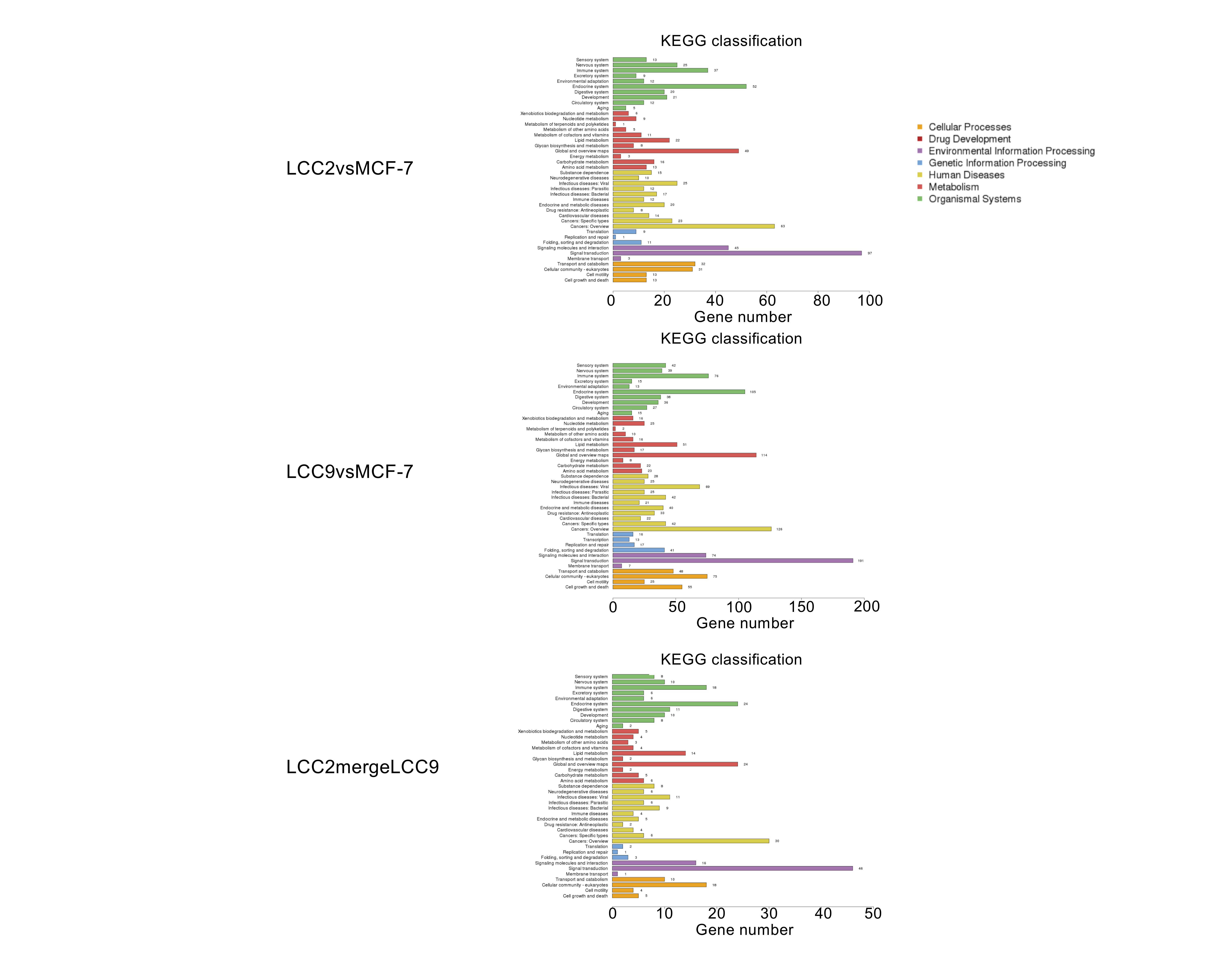

Supplement: Supplementary file 2 [file DataSheet_2.zip › Supplementary Figures_1/Supplementary Figure 4.JPEG]

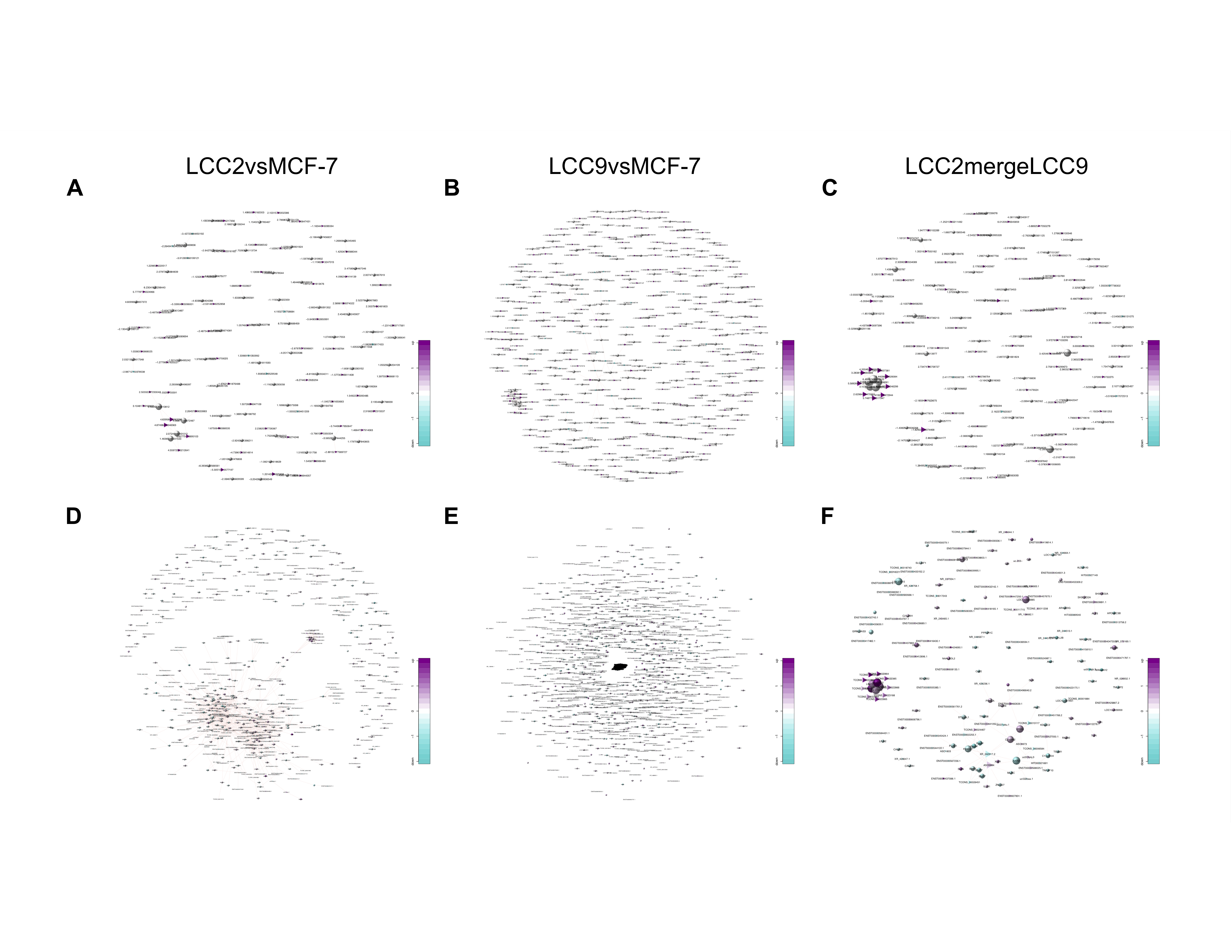

Supplement: Supplementary file 2 [file DataSheet_2.zip › Supplementary Figures_1/Supplementary Figure 5.JPEG]

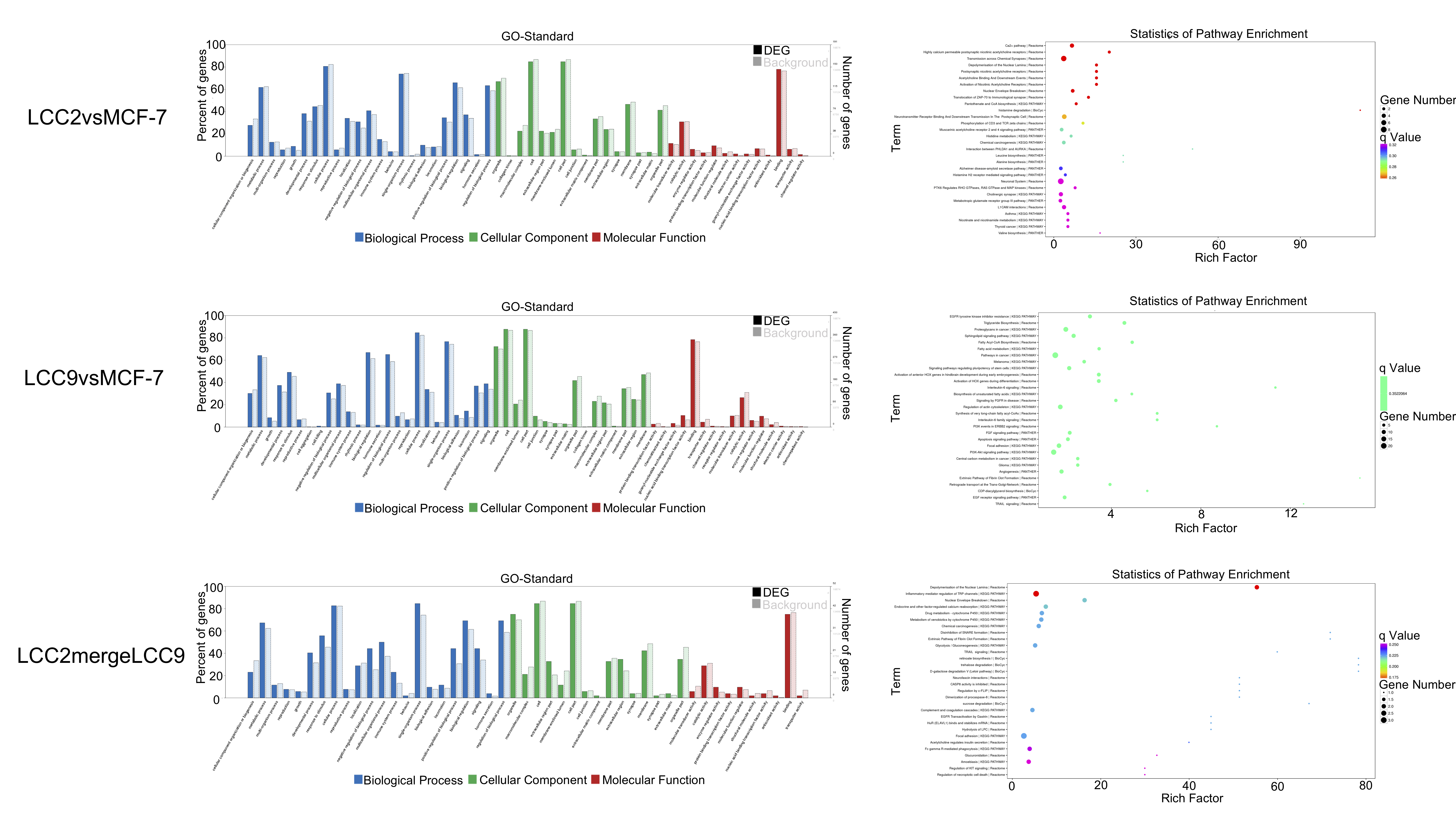

Supplement: Supplementary file 2 [file DataSheet_2.zip › Supplementary Figures_1/Supplementary Figure 6.JPEG]

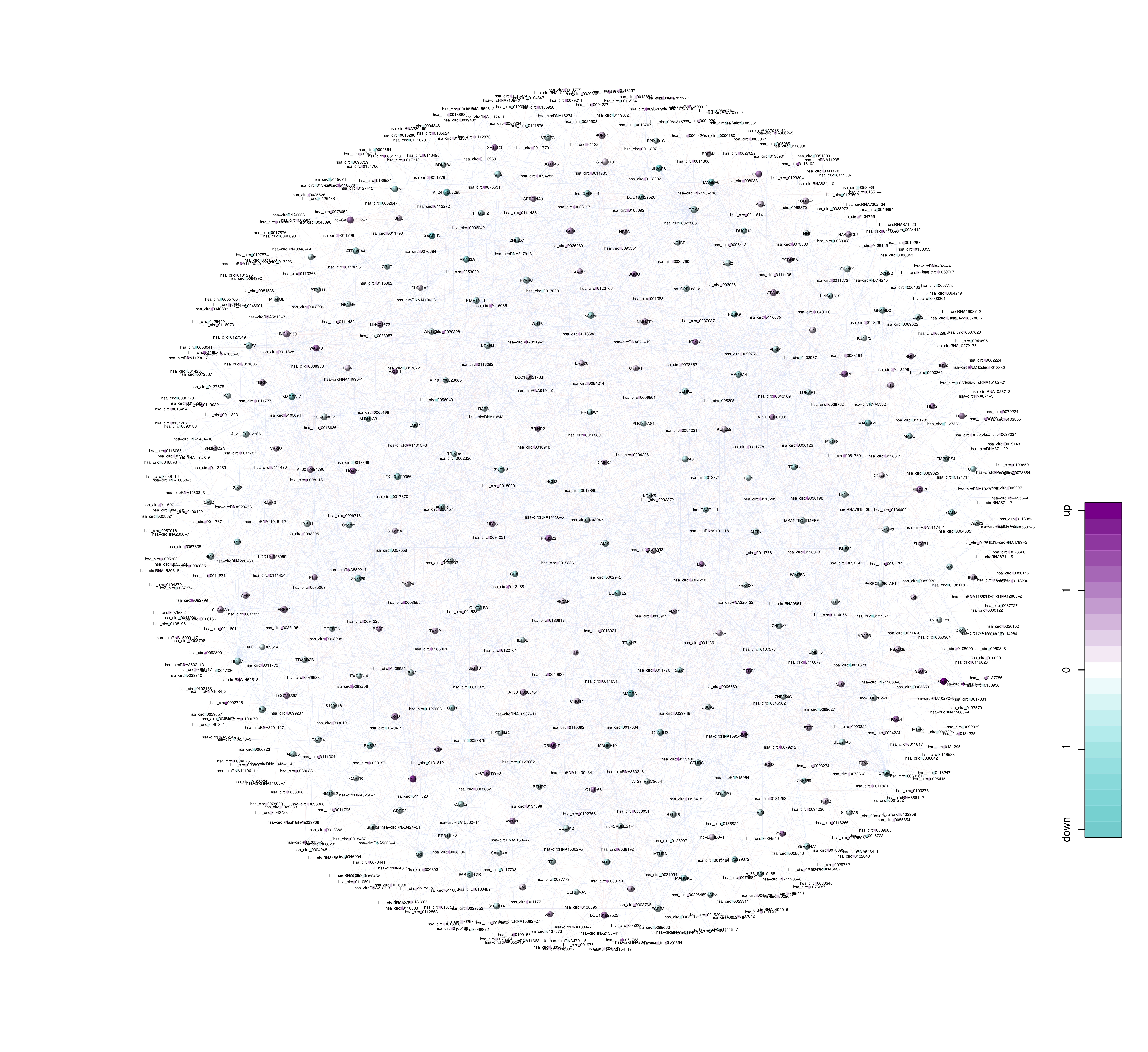

Supplement: Supplementary file 2 [file DataSheet_2.zip › Supplementary Figures_1/Supplementary Figure 7.JPEG]

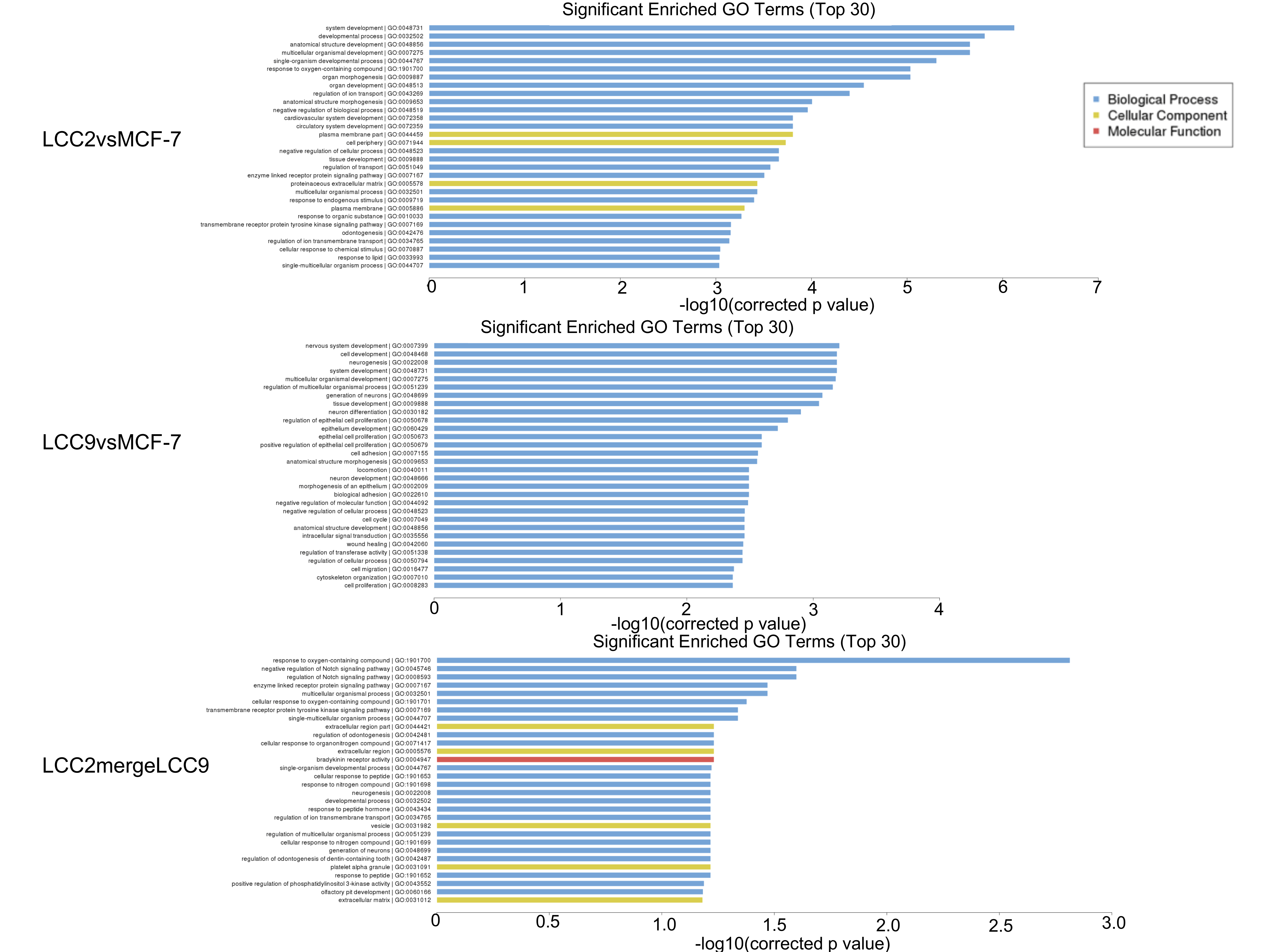

Supplement: Supplementary file 2 [file DataSheet_2.zip › Supplementary Figures_1/Supplementary Figure 8.JPEG]

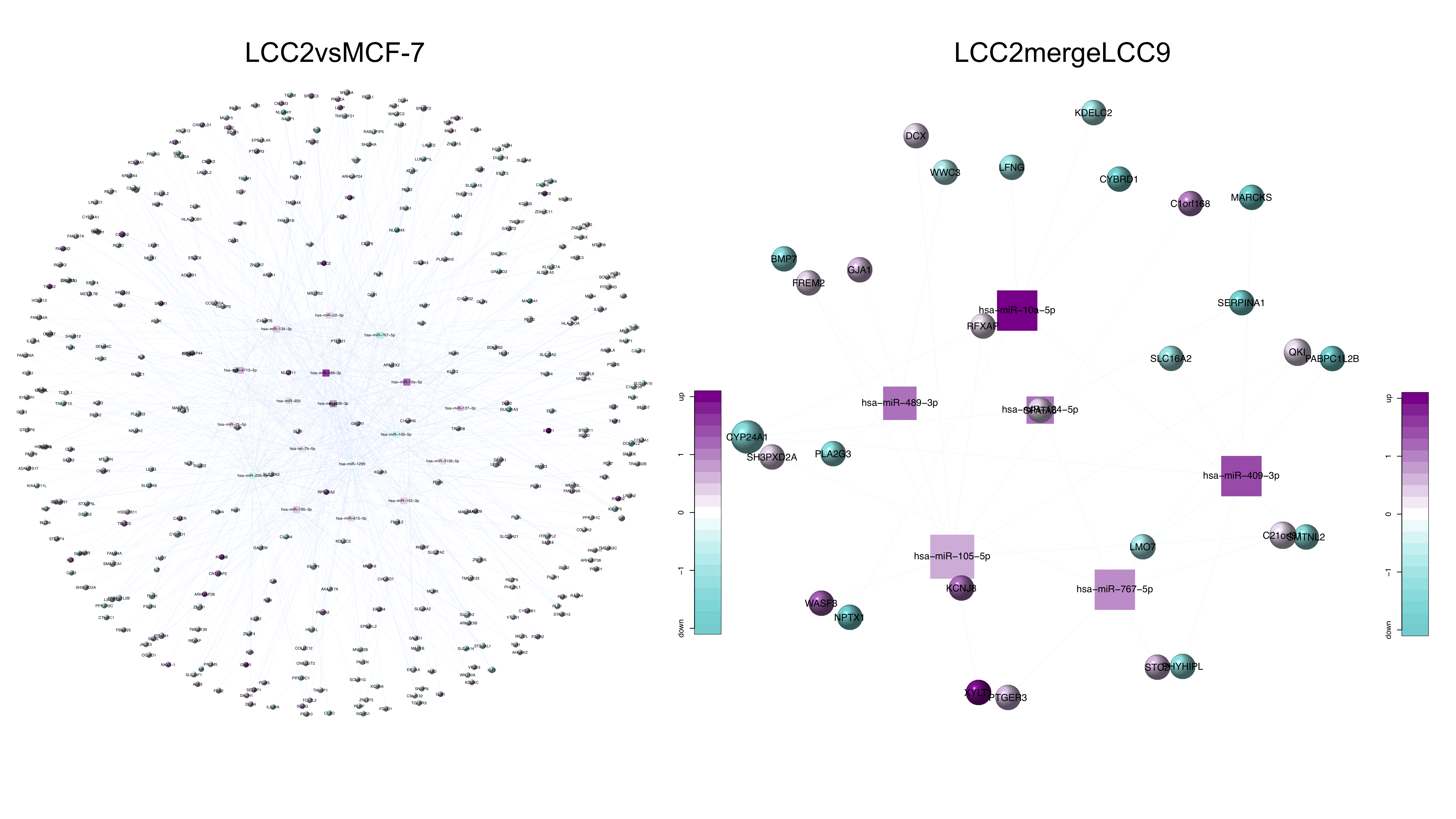

Supplement: Supplementary file 3 [file DataSheet_3.zip › Supplementary Figures_2/Supplementary Figure 10.JPEG]

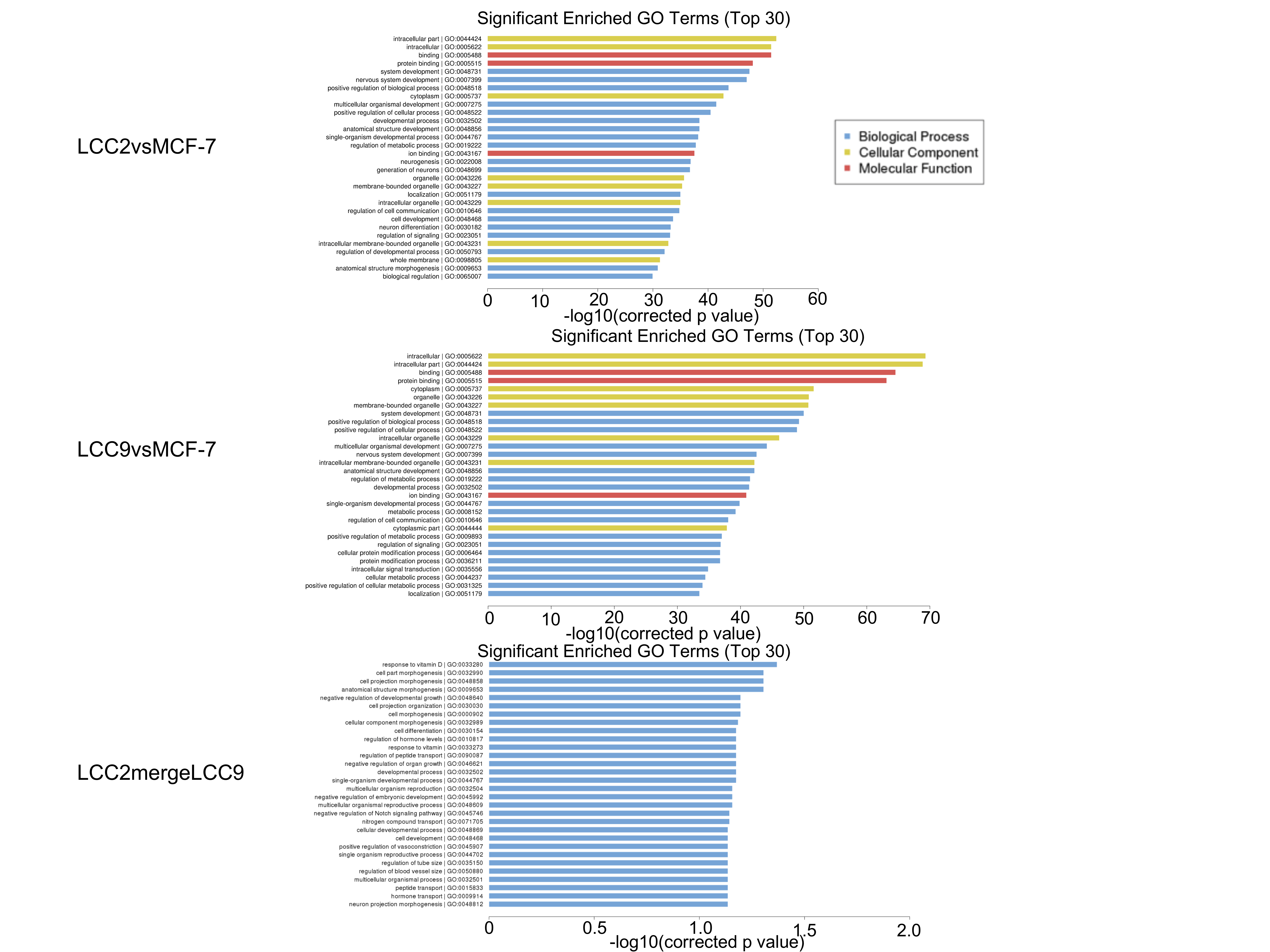

Supplement: Supplementary file 3 [file DataSheet_3.zip › Supplementary Figures_2/Supplementary Figure 11.JPEG]

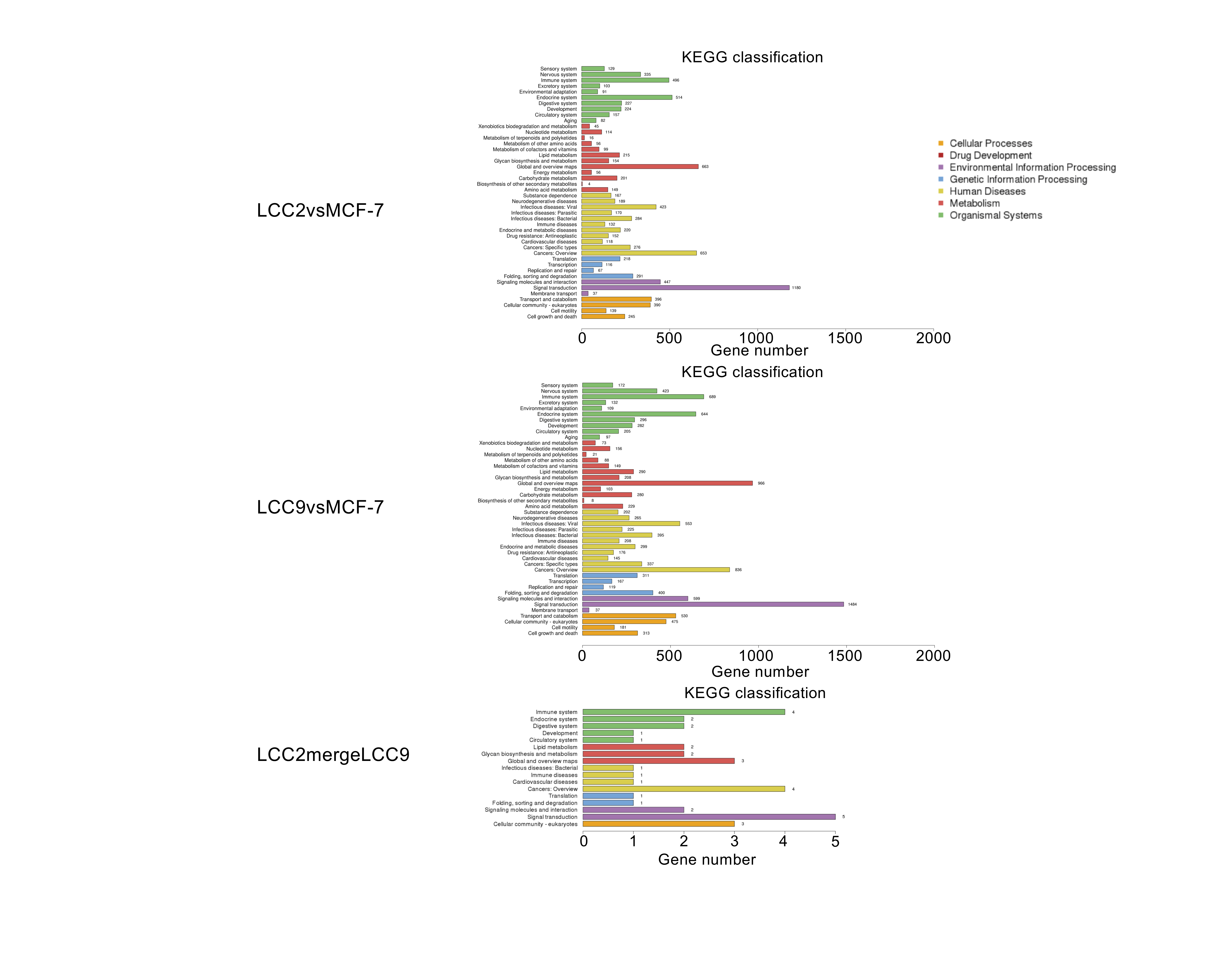

Supplement: Supplementary file 3 [file DataSheet_3.zip › Supplementary Figures_2/Supplementary Figure 12.JPEG]

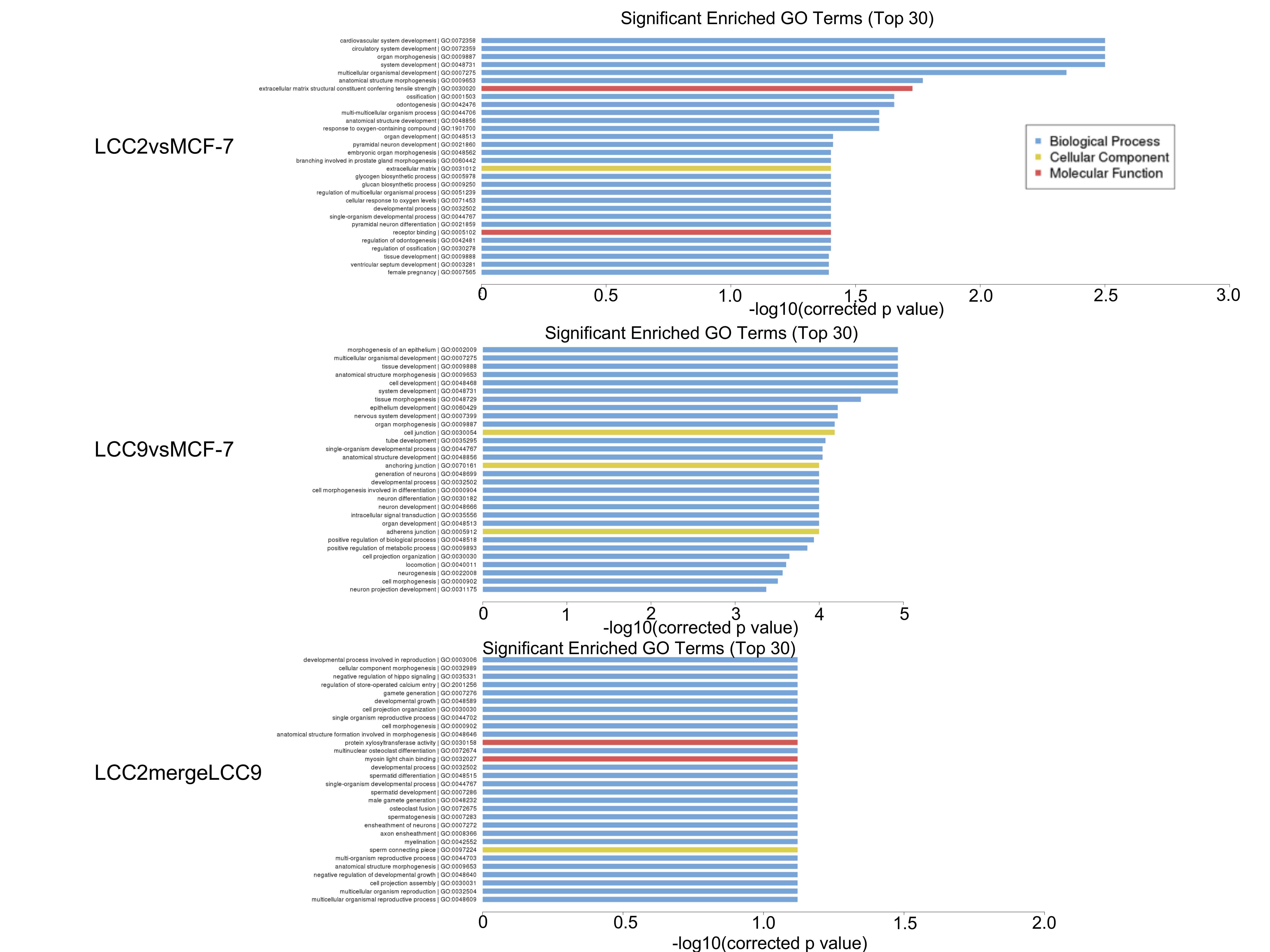

Supplement: Supplementary file 3 [file DataSheet_3.zip › Supplementary Figures_2/Supplementary Figure 13.JPEG]

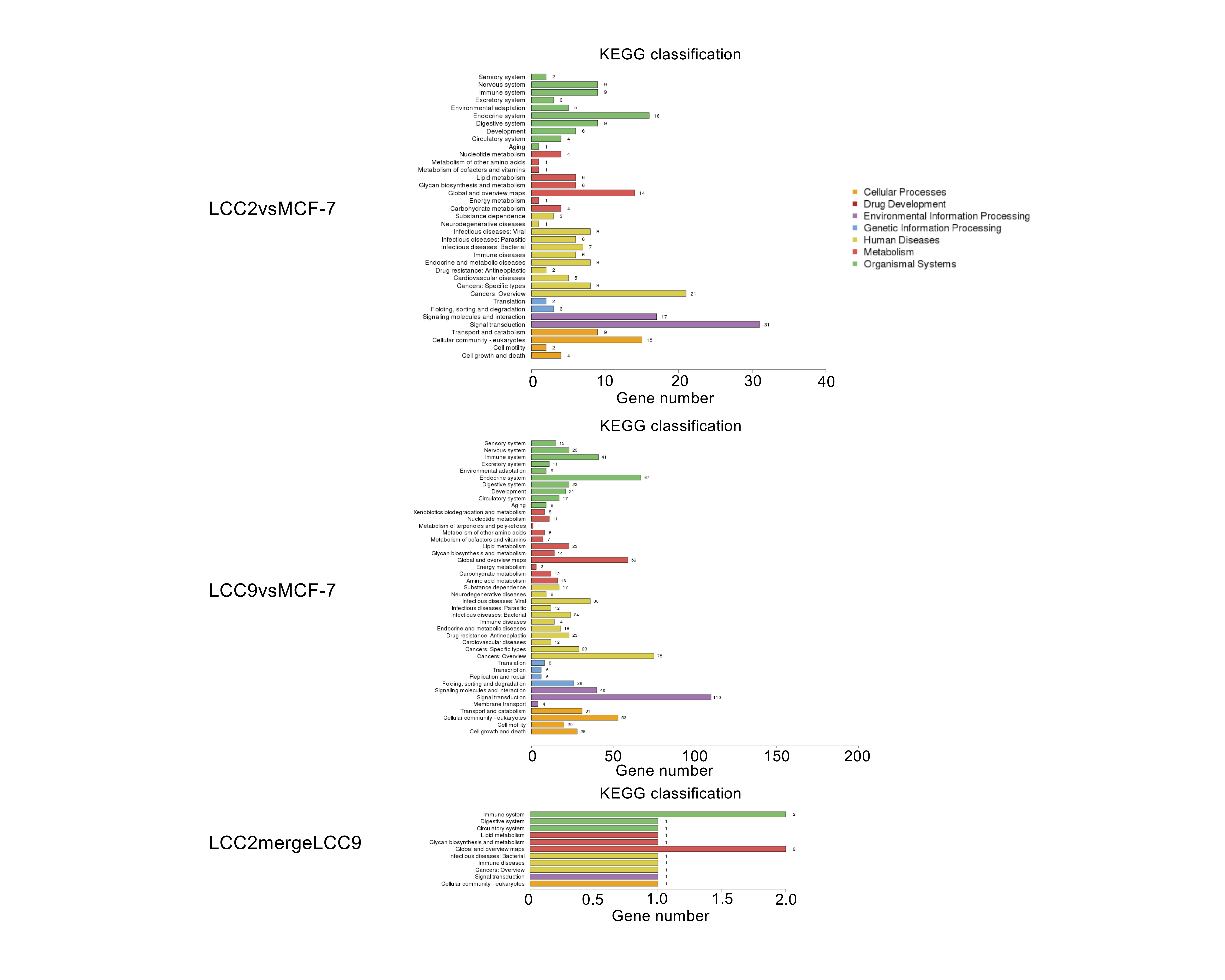

Supplement: Supplementary file 3 [file DataSheet_3.zip › Supplementary Figures_2/Supplementary Figure 14.JPEG]

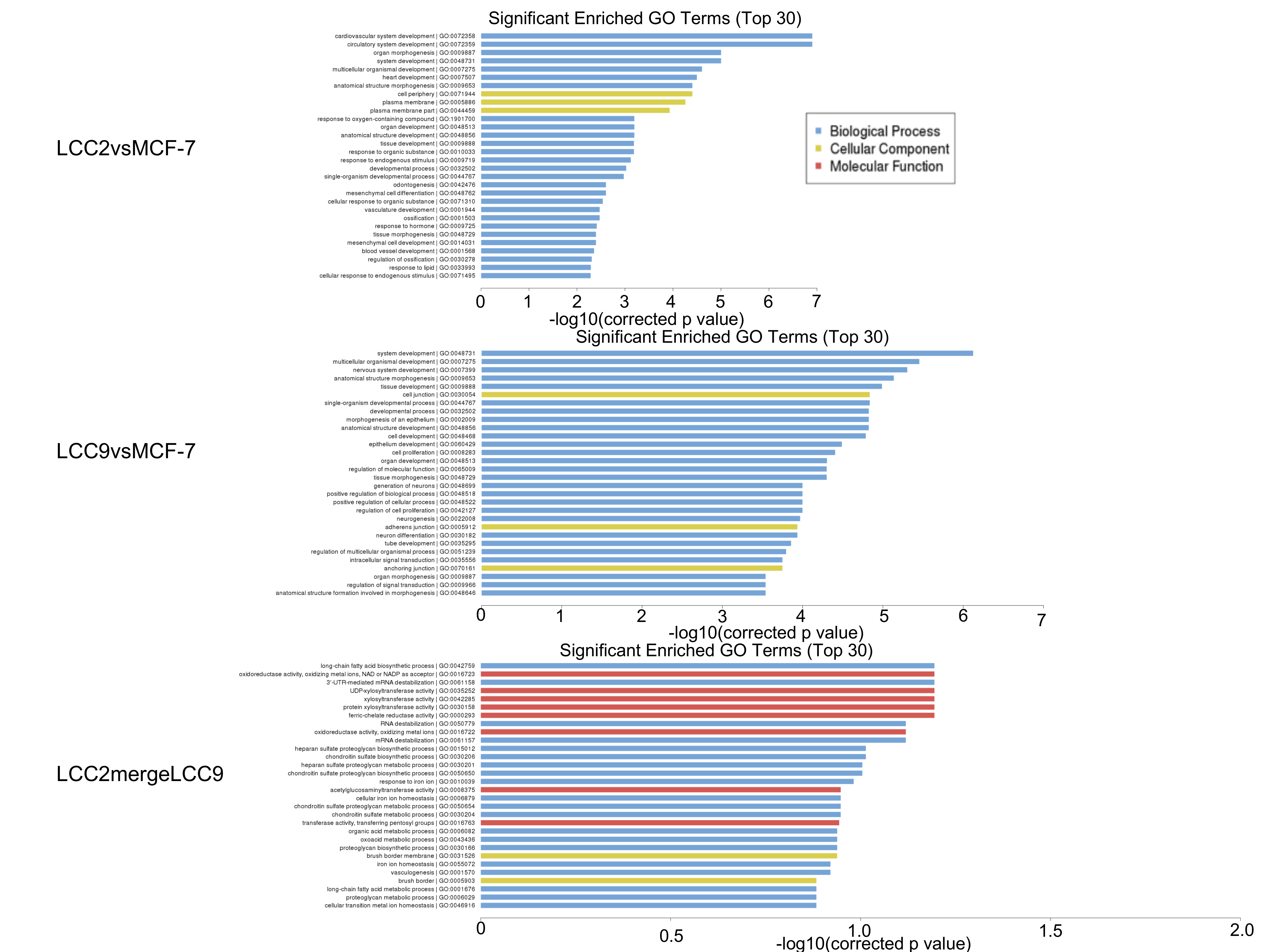

Supplement: Supplementary file 3 [file DataSheet_3.zip › Supplementary Figures_2/Supplementary Figure 15.JPEG]

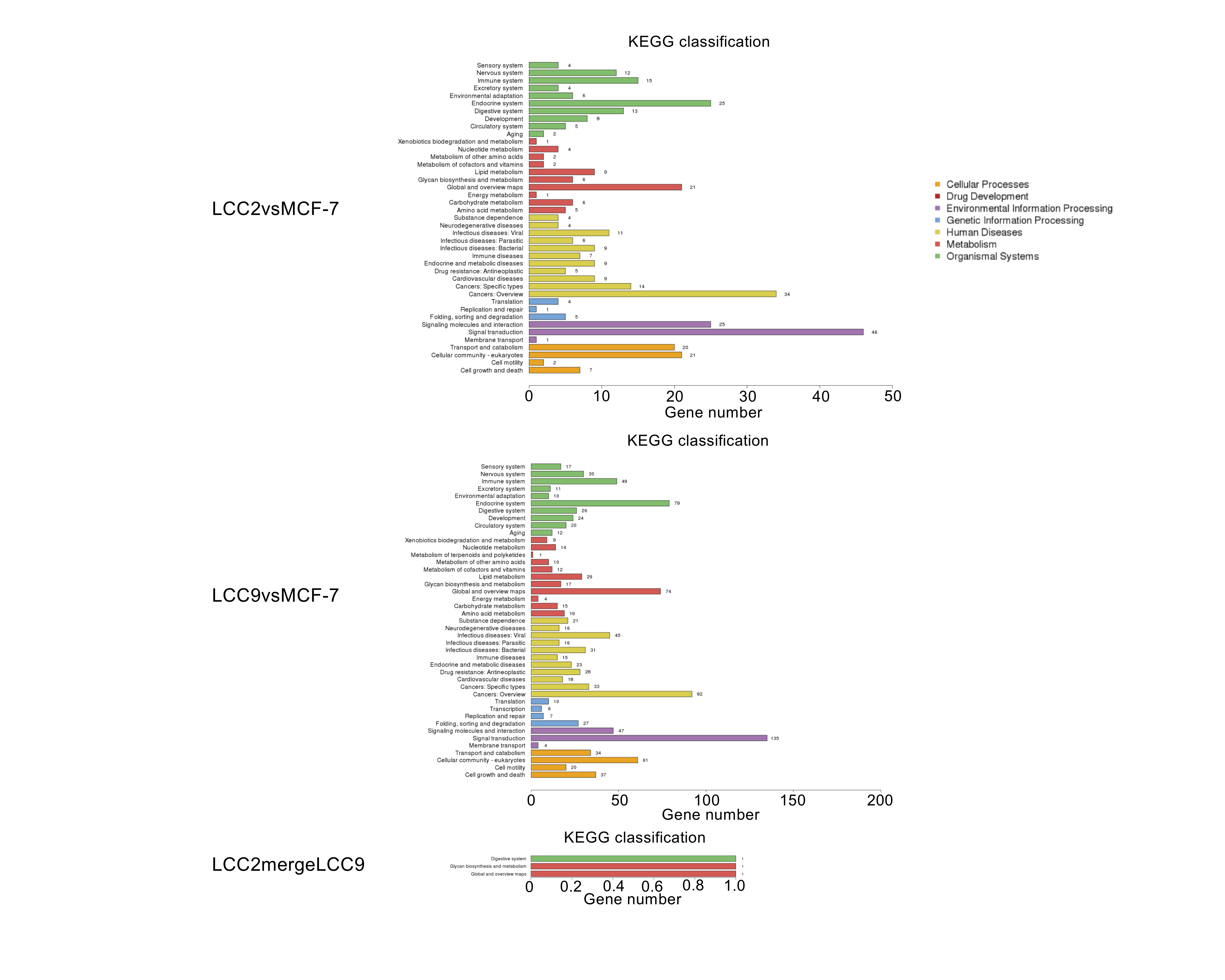

Supplement: Supplementary file 3 [file DataSheet_3.zip › Supplementary Figures_2/Supplementary Figure 16.JPEG]

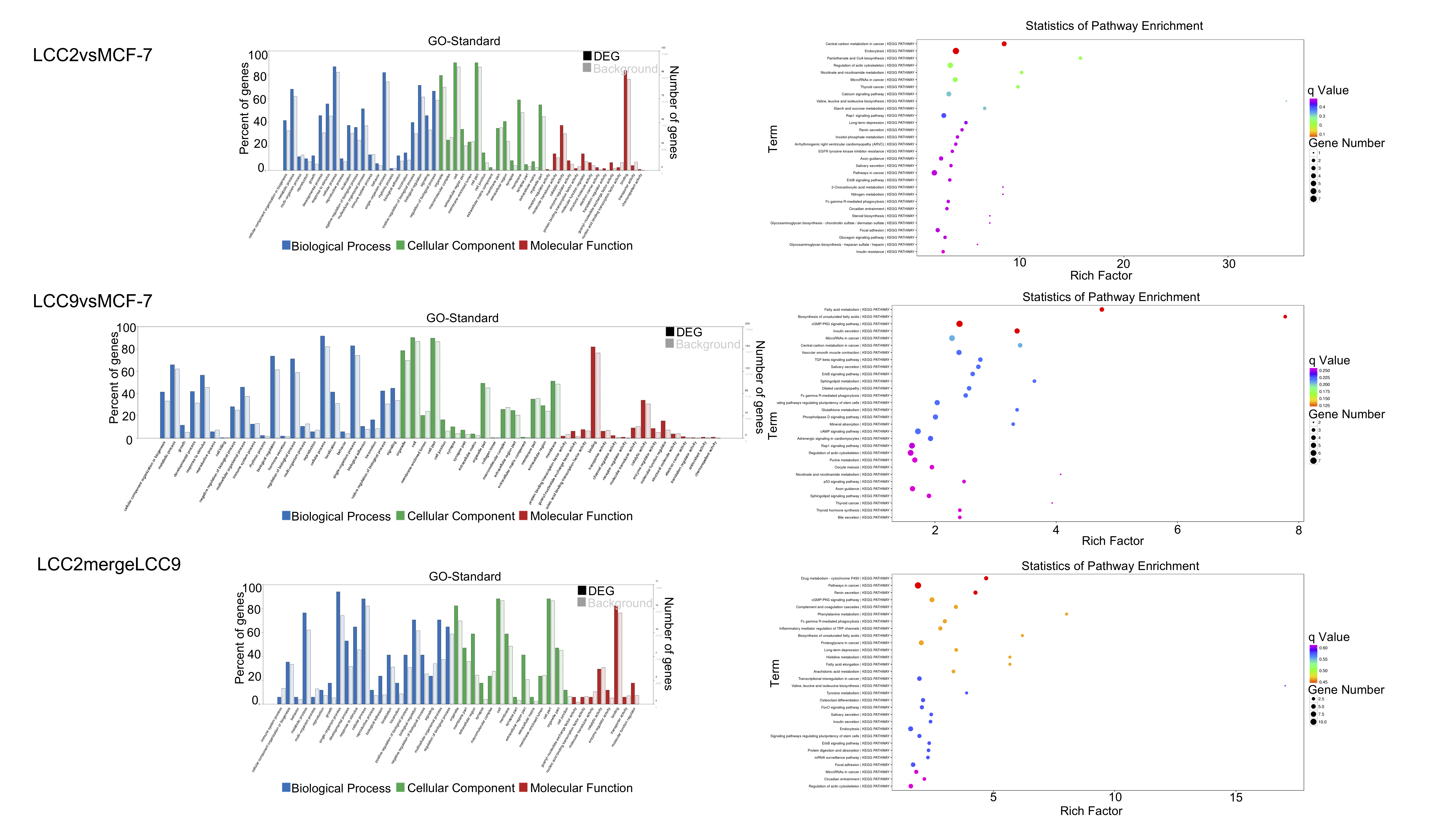

Supplement: Supplementary file 3 [file DataSheet_3.zip › Supplementary Figures_2/Supplementary Figure 17.JPEG]

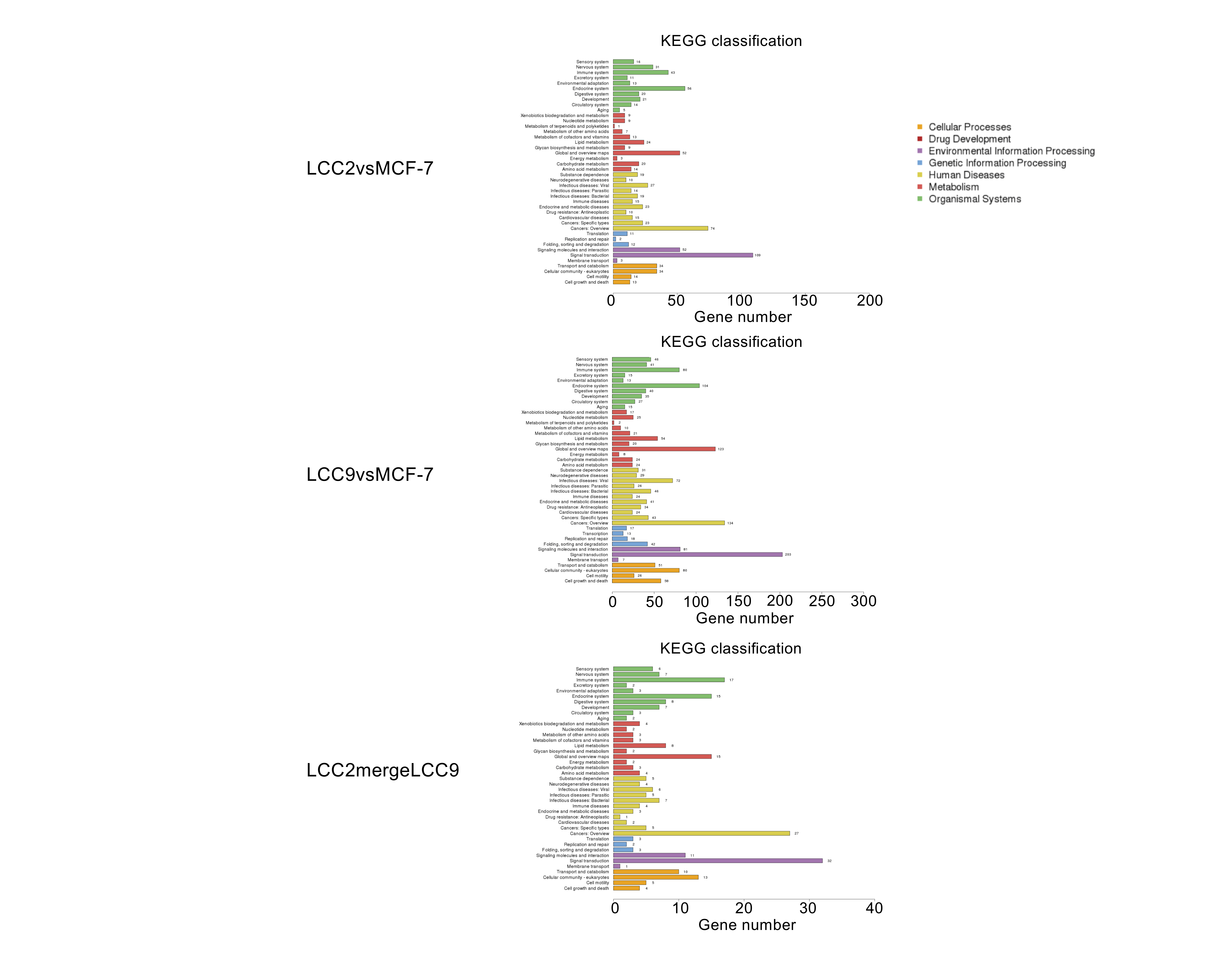

Supplement: Supplementary file 3 [file DataSheet_3.zip › Supplementary Figures_2/Supplementary Figure 9.JPEG]
